# Tissue block-resolved developmental transcriptomic atlas of human fetal brainstem reveals gene modules with implications for neurological disorders

**DOI:** 10.3389/fcell.2025.1674967

**Published:** 2025-11-28

**Authors:** Chengxin Liu, Wenjuan Zhou, Xin Xing, Jiachen Chen, Chenxi Sun, Shizhou Liu, Yunxia Lou, Jianfen Jiao, Haoling Cao, Baoxia Cui, Shuhui Hong, Niloufar Ahmadi, Yuchun Tang

**Affiliations:** 1 Department of Anatomy and Neurobiology, Key Laboratory of Experimental Teratology of the Ministry of Education, Shandong Key Laboratory of Mental Disorders and Intelligent Control, Shandong Key Laboratory of Digital Human and Clinical Anatomy, School of Basic Medical Sciences, Cheeloo College of Medicine, Institute for Sectional Anatomy and Digital Human, Shandong University, Jinan, Shandong, China; 2 Institute of Brain and Brain-Inspired Science, Shandong University, Jinan, Shandong, China; 3 Department of Obstetrics and Gynecology, The First Affiliated Hospital of Shandong First Medical University and Shandong Provincial Qianfoshan Hospital, Jinan, Shandong, China; 4 Department of Ultrasound, Qilu Hospital of Shandong University, Jinan, Shandong, China; 5 Department of Obstetrics and Gynecology, Qilu Hospital of Shandong University, Jinan, Shandong, China

**Keywords:** fetal gene expression, fetal development, brainstem, RNA sequencing, gene regulation

## Abstract

**Introduction:**

The brainstem is a critical hub connecting the cerebrum and spinal cord. However, the gene regulatory dynamics during fetal brainstem development remain incompletely characterized.

**Methods:**

This study employed RNA-seq to map transcriptomes across 107 tissue blocks from 18 human fetal brainstems (gestational weeks 9–33). Weighted gene co-expression network analysis (WGCNA) identified 22 functionally annotated modules. We quantitatively assessed their spatiotemporal activity gradients and systematically classified genes exhibiting significant temporal trajectories based on phase-specific signatures.

**Results:**

Our integrated approach constructed a developmental transcriptomic profile, revealing stage-specific regulatory networks and dynamic transcriptional trajectories governing ontogeny. Crucially, we validated the expression of neurodevelopmental disorder-associated genes within fetal brainstem tissues.

**Discussion:**

This work advances our understanding of brainstem development and provides a foundational resource for research into neurological disorders.

## 1 Introduction

The brainstem is a pivotal neural structure that serves as the primary interface between the cerebrum and spinal cord, playing an indispensable role in regulating fundamental physiological processes. In addition to its primary regulatory functions in vital processes including respiratory control, cardiovascular modulation, and blood pressure homeostasis (Guyenet and Bayliss, 2015), the brainstem serves as the central hub for autonomic nervous system regulation. The structural complexity of this region, characterized by its intricate neural networks and diverse nuclei, is essential for the maintenance of systemic homeostasis in the organism.

Growing evidence demonstrates significant associations between brainstem abnormalities and various neuropsychiatric disorders. Parkinson’s disease, for instance, is characterized by substantial degeneration of dopaminergic neurons in the substantia nigra of the brainstem (Dauer and Przedborski, 2003). Additionally, the pathophysiology of depression, anxiety disorders, and schizophrenia may involve neurotransmitter imbalances or structural abnormalities in brainstem regions (Morris et al., 2020). Given its role as a component of the cerebellar system (encompassing the Guillain-Mollaret triangle), the origin site for most cranial nerves, and a critical conduit for ascending and descending axonal tracts linking the forebrain and spinal cord, the brainstem is intricately involved in a spectrum of cerebellar malformations. These include Chiari malformation type II, rhombencephalosynapsis, mesencephalosynapsis, Dandy-Walker spectrum disorders, and pontocerebellar hypoplasia. Brainstem-related disorders are associated with diverse manifestations of developmental anomalies or functional impairments, underscoring the importance of elucidating normal brainstem development for disease prevention and therapeutic interventions.

While significant progress has been made in mapping gene expression in cerebral regions, particularly through single-cell sequencing of the telencephalon (Li et al., 2023), the brainstem remains relatively underexplored. Its complex architecture (Tang et al., 2018), diverse functional roles, and unique developmental morphology contribute to persistent knowledge gaps regarding the spatiotemporal regulation of gene expression and the molecular mechanisms governing its ontogeny. Consequently, a comprehensive characterization of gene regulation across the entire fetal brainstem lags behind that of other brain regions.

The development of the brainstem during fetal central nervous system formation represents a critical phase in neurogenesis. This process is meticulously orchestrated by key regulatory genes, including the human fetal rhombomere homeobox genes (such as the Hox family), which act as master regulators to define the segmental identity and patterning of the hindbrain regions that give rise to the brainstem (Prince et al., 1998). As a structure derived from the midbrain-hindbrain region, the brainstem undergoes these complex ontogenetic processes that are particularly susceptible to genetic mutations. Disruptions in the precise expression of these genes can result in significant structural malformations and various neuropsychiatric disorders.

During embryonic development, the brainstem undergoes significant morphological and functional transformations, and its involvement in various neurological disorders, including amyotrophic lateral sclerosis (ALS) (Tahedl et al., 2024), Autism Spectrum Disorder (ASD) (Connacher et al., 2022),Parkinson’s disease (PD) (Xiong et al., 2021), and Alzheimer’s disease (AD) (Beldean et al., 2025), is well documented. These disorders are closely associated with specific gene expression patterns and molecular interactions during brainstem development. To delineate the gestational age-dependent expression trajectories of neurodevelopmental and neurodegenerative disease-associated genes in the developing human brainstem, transcriptomic profiling with extensive temporal sampling (spanning critical developmental windows) and spatial resolution across anatomically defined subregions is methodologically indispensable. RNA sequencing (RNA-seq) was conducted on anatomically partitioned subregions (midbrain, pons, and medulla oblongata) of brainstem tissues obtained from 18 fetal specimens spanning gestational weeks 9–33. Through systematic analysis of gene expression patterns, a comprehensive developmental transcriptomic profile was constructed to characterize the molecular dynamics underlying human fetal brainstem development. Our sampling covered major developmental periods, including pivotal stages, with precision enhanced through anatomical subregion segmentation. We utilized this resource to analyze developmental gene modules and expression changes, annotate neuropsychiatric disorder-associated genes, identify genes critical for physiological functions, and validate protein expression through immunofluorescence. This comprehensive transcriptomic profile provides a pivotal resource for deciphering the molecular foundations of brainstem-mediated vital functions and establishes an essential framework for investigating the developmental origins of brainstem-associated neuropathologies.

## 2 Materials and methods

### 2.1 Sample acquisition

All samples were obtained from the Brain Bank of Shandong University. The human fetal collection and research protocols were approved by the Ethics Committee of Shandong University (ECSBMSSDU2024-1-9). Specimens spanning various gestational ages—calculated based on the last menstrual period—with intact morphology and minimal preservation duration were carefully selected from the biobank. All fetal samples were collected within 2 h after abortion for subsequent sequencing analysis. Brainstem samples from 18 human fetuses (gestational ages 9–33 weeks) were used for RNA-seq, with an additional sample from a 24-week fetus for immunofluorescence. Additional information, including fetal sex, has been provided in the Supplementary Material.

### 2.2 Sample storage

The brainstem tissues used for RNA-seq were collected from fetal specimens within 2 h after abortion or induced labor, rapidly frozen in liquid nitrogen, and subsequently stored in a −80 °C freezer (Haier, DW-86L626).

### 2.3 Tissue sampling

Precise microdissection of the human fetal brainstem was performed using a dissection microscope (Model NSZ-810, Ningbo Yongxin Optics Co., Ltd.) and fine anatomical tools, with reference to human fetal brain atlas for anatomical guidance. The frozen brainstem samples, collected from fetuses of varying gestational ages and stored at −80 °C, were partially thawed on a chilled plate to maintain RNA integrity. Under the microscope, distinct regions within the brainstem were carefully identified. Using sterile, RNAse-free micro-scalpels and forceps, specific tissue blocks (approximately 3 mg each) were isolated from these targeted locations. Throughout the procedure, all instruments and surfaces were treated with RNase decontamination solutions to prevent RNA degradation. The collected tissue blocks were immediately transferred to RNase-free microtubes and stored at −80 °C until subsequent RNA extraction and RNA-seq library preparation.

### 2.4 RNA sequencing

#### 2.4.1 RNA extraction

Total RNA was extracted from tissues using TRIzol (Invitrogen, Carlsbad, California, USA) according to manual instruction.

Appropriate amount of tissue was ground into powder under liquid nitrogen, then transferred into a 2 mL EP tube containing 1.5 mL of TRIzol, standing for 5 min. For cell samples, the TRIzol was directly added to the cell sample with the amount 1 mL per 1 × 10^6^ cells, followed by vortex mixing and standing for 5-min. The mixture was centrifuged at 4 °C at 12,000 x g for 5 min, and the supernatant was transferred to a new EP tube for extraction. 300 μL of chloroform/isoamyl alcohol (24:1) was added into the transferred supernatant and the mixture was vigorously vortexed to ensure thorough mixing. Then the mixture was centrifuged at 4 °C and 12,000 x g for 8 min. Take the clear upper aqueous layer and repeat the above process one time. After centrifugation, the final upper aqueous layer was carefully transferred to a new 1.5 mL EP tube with a 2/3 supernatant volume of isopropanol, gently inverted to mix well, placed in a −20 °C refrigerator for 2 h.

Next, the precipitation mixture was centrifuged with 17,500 x g at 4 °C for 25 min. The supernatant was discarded, and the precipitation was washed with 0.9 mL of 75% ethanol. The precipitation was suspended by inverting the tube up and down several times. Then the precipitation was collected by centrifuging at 17500 g for 3 min at 4 °C and discarding the supernatant. Repeat the centrifugation to remove supernatant completely and the precipitation was dried in the biosafety cabinet for 3–5 min. Finally, 20 µL∼200 µL of DEPC-treated or RNase-free water was added to dissolve the RNA. Subsequently, total RNA was qualified and quantified using a Fragment Analyzer or Agilent 2100 Bioanalyzer (Agilent, CA, USA), or Qseq-400 (Bioptic, Taiwan, China). Samples with a RIN >6 were used for library preparation.

#### 2.4.2 mRNA library preparation

Library preparation is performed using Optimal Dual-mode mRNA Library Prep Kit (BGI-Shenzhen, China). A certain amount of RNA are denatured at suitable temperature to open the secondary structure, and mRNA is enriched by oligo (dT) attached magnetic beads. After reacting at a suitable temperature for a fixed time period, RNAs are fragmented with fragmentation reagents.

Then First-strand cDNA is generated using random hexamer-primed reverse transcription, followed by a second-strand cDNA synthesis. The synthesized double strand cDNA is subject to end repairment reaction. After cDNA end repairment, a single “A” nucleotide is added to the 3′ ends of the blunt fragments through A tailing reaction. Then the reaction system for adaptor ligation configured to ligate adaptors with the cDNAs, and finally, the library products are amplified through PCR reaction and subjected to quality control.

Next, the single-stranded library products are produced via denaturation. The reaction system for circularization is set up to get the single-stranded cyclized DNA products. Any uncyclized single stranded linear DNA molecules will be digested. The final single strand circularized library is amplified with phi29 and rolling circle amplification (RCA) to make DNA nanoball (DNB) which carries more than 300 copies of the initial single stranded circularized library molecule. The DNBs are loaded into the patterned nanoarray and PE 150 bases reads are generated on T7 platform (BGI-Shenzhen, China).

#### 2.4.3 Data filtering

The sequencing data was filtered with SOAPnuke (Li et al., 2008) by (1) Removing reads containing sequencing adapter; (2) Removing reads whose low-quality base ratio (base quality less than or equal to 15) is more than 20%; (3) Removing reads whose unknown base (“N” base) ratio is more than 5%, afterwards clean reads were obtained and stored in FASTQ format. The subsequent analysis and data mining were performed on Dr. Tom Multi-omics Data mining system (https://biosys.bgi.com).

#### 2.4.4 Structure variation detection

The clean reads were mapped to the reference genome using HISAT2 (Kim et al., 2015) against the *Homo sapiens* GCF_000001405.39_GRCh38.p13 assembly (NCBI). Only samples with a mapping rate greater than 95% were subjected to further analysis. After that, Ericscript (v0.5.5) (Benelli et al., 2012) and rMATS (V4.1.2) (Shen et al., 2014) were used to detect fusion genes and differential splicing genes (DSGs).

#### 2.4.5 RNA identification

Bowtie2 (Langmead and Salzberg, 2012) was applied to align the clean reads to the gene set, in which known and novel, coding and noncoding transcripts were included.

#### 2.4.6 Gene quantification differential expression analysis

Expression level of gene was calculated by RSEM (v1.3.1) (Li and Dewey, 2011). The heatmap was drawn by pheatmap (v1.0.12) according to the gene expression difference in different samples. Differential expression analysis was primarily performed using DESeq2 (v1.34.0) (Love et al., 2014). P-values were generated using the Wald test and adjusted for multiple testing using the Benjamini–Hochberg procedure. Genes with an adjusted p-value (FDR) ≤ 0.05 and absolute log2 fold change ≥1 were considered significantly differentially expressed. Essentially, differential expression analysis was performed using the DESeq2 (v1.34.0) (or DEGseq (v1.48.0) (Wang et al., 2010) or PoissonDis (Audic and Claverie, 1997)) with Q value ≤0.05 (or FDR ≤0.001).

### 2.5 WGCNA

WGCNA was performed on 18 embryonic brainstem samples using the WGCNA package (Langfelder and Horvath, 2008) in R (version 4.3.2). Module eigengenes and trait associations were calculated using Pearson correlation. Based on the topological overlap matrix (TOM) derived from a pairwise correlation-based adjacency matrix, the topological overlap similarity among genes was calculated. The criterion for selecting the soft threshold was to choose the minimum power value at which the scale-free topology fit index (SFT.R.sq) first reached or exceeded 0.90. Gene co-expression modules were subsequently identified through average linkage hierarchical clustering and labeled with distinct colors. The Dynamic Hybrid Tree Cut algorithm (Langfelder et al., 2008), which detects clusters in a dendrogram by adaptively combining static height-based cutting with dynamic branch cutting based on shape, was employed to define gene co-expression modules, resulting in the identification of 22 distinct modules.

### 2.6 GSVA

Gene set variation analysis (GSVA)was implemented using the GSVA R package (version 4.3.2) to calculate enrichment scores reflecting pathway-level activity of gene sets, which were identified via WGCNA. The GSVA algorithm transformed gene expression matrices into pathway-centric score matrices, enabling unsupervised quantification of inter-sample biological functional variation. Enrichment scores for each sample-pathway feature pair were computed through a non-parametric approach, establishing a data foundation for subsequent analyses of pathway dysregulation in relation to molecular subtypes or phenotypic traits (Hänzelmann et al., 2013).

### 2.7 Mfuzz pseudo-timing analysis

Gene expression profiles from embryos at five distinct gestational weeks were clustered using the fuzzy c-means algorithm implemented in the Mfuzz package (version R4.3.2) (Kumar and Futschik, 2007) within R.

### 2.8 Statistical analysis

KEGG pathway analysis, GO enrichment analysis and data visualization were conducted with the Dr. Tom program (https://biosys.bgi.com). A p-value or adjusted p-value (FDR/Q value) < 0.05 was considered statistically significant.

### 2.9 Immunofluorescence microscopy analysis

#### 2.9.1 Sample fixation

1. Brain Tissue Harvesting: Brainstem tissues were carefully dissected from a 24-week gestational age specimen.
2. Formalin Fixation: The brain tissues were immersed in 4% paraformaldehyde (pH 7.4), which facilitated subsequent tissue sectioning procedures.

#### 2.9.2 Dehydration and sucrose infiltration

Following fixation, the brain tissues were dehydrated using a graded series of ethanol solutions to remove water content, thereby facilitating subsequent cryosectioning. The dehydration protocol was as follows.

70% ethanol: 5 min.
80% ethanol: 2 h.
90% ethanol: 2 h.
95% ethanol: Two changes, 2 h each.
100% ethanol (absolute ethanol): Two changes, 2 h each.

#### 2.9.3 Sucrose infiltration

For the first sucrose infiltration, the brain tissues were immersed in a 30% sucrose solution for 72 h to ensure complete dehydration and to allow the tissues to sink. After the first infiltration, the specimens were transferred to a fresh 30% sucrose solution for an additional 48 h to ensure complete tissue saturation and sinking.

#### 2.9.4 Freezing and embedding

The brain tissues were embedded using optimal cutting temperature (OCT) compound and subsequently stored at −20 °C for 24 h to ensure tissue stability during the sectioning process.

#### 2.9.5 Sectioning and storage

1. Section Preparation: Prior to sectioning, the cryostat was pre-cooled to −20 °C to ensure optimal sectioning quality.
2. Sectioning Procedure: The embedded brain tissues were placed in the cryostat, and the blade and anti-roll plate were carefully aligned. Serial sections of 10 μm thickness were cut and gently transferred onto glass slides.
3. Section Storage: The prepared brain tissue sections were stored at −20 °C for long-term cryopreservation.

#### 2.9.6 Immunofluorescence microscopy

The tissue sections were subjected to antigen retrieval with citric acid (PH6.0) antigen retrieval buffer and washed with PBS three times. 3% BSA was used in blocking for 1 h at room temperature, and then incubated with primary antibodies overnight at 4 °C. Secondary antibodies were incubated at room temperature for 50 min. Sections were then counterstained with DAPI (Sigma, Cat#F6057). The stained sections were imaged on TissueFaxs Spectra (TissueGnostics, Austria). The antibodies used are listed in Supplementary Table S6.

1. Fixation of Cryosections: The cryosections were dried in a 37 °C oven for 20 min to remove moisture. Subsequently, the sections were fixed with 4% paraformaldehyde for 30 min, followed by three washes in phosphate-buffered saline (PBS, pH 7.4) on a decolorizing shaker, 5 min each.
2. Antigen Retrieval: The tissue sections were placed in a retrieval box filled with EDTA antigen retrieval buffer (pH 8.0) and subjected to microwave-mediated antigen retrieval. The retrieval protocol was as follows: medium-low power for 8 min, followed by an 8-min resting period, and then low power for 7 min. During this process, care was taken to prevent excessive evaporation of the buffer and to avoid drying of the sections. After natural cooling, the slides were washed three times in phosphate-buffered saline (PBS, pH 7.4) on a decolorizing shaker, 5 min each.
3. Circular Barrier and Serum Blocking: After gently removing excess liquid from the sections, a hydrophobic barrier was drawn around the tissues using a histochemical pen to prevent antibody runoff. The remaining PBS was then removed, and the sections were incubated with bovine serum albumin (BSA) for 30 min for blocking.
4. Primary Antibody Incubation: After gently removing the blocking solution, the sections were incubated with primary antibodies diluted in phosphate-buffered saline (PBS) at the appropriate ratio. The slides were placed horizontally in a humidified chamber and incubated overnight at 4 °C. (A small amount of water was added to the chamber to prevent evaporation of the antibody solution.)
5. Secondary Antibody Incubation: The slides were washed three times in phosphate-buffered saline (PBS, pH 7.4) on a decolorizing shaker, 5 min each. After gently removing excess PBS, the sections were incubated with fluorescence-conjugated secondary antibodies corresponding to the species of the primary antibodies. The secondary antibodies were applied dropwise to cover the tissues within the hydrophobic barrier, and the slides were incubated in the dark at room temperature for 50 min.
6. DAPI Nuclear Staining: The slides were washed three times in phosphate-buffered saline (PBS, pH 7.4) on a decolorizing shaker, 5 min each. After gently removing excess PBS, DAPI staining solution was applied dropwise to cover the tissues within the hydrophobic barrier. The slides were then incubated in the dark at room temperature for 10 min.
7. Quenching of Tissue Autofluorescence: The slides were washed three times in phosphate-buffered saline (PBS, pH 7.4) on a decolorizing shaker, 5 min each. Autofluorescence quenching reagent was applied dropwise within the hydrophobic barrier and incubated for 5 min. Subsequently, the slides were rinsed under running water for 10 min.
8. Mounting:After gently removing excess liquid, the sections were mounted using an anti-fade mounting medium and coverslips to prevent fluorescence quenching.
9. Microscopy and Image Acquisition: The sections were observed under a fluorescence microscope, and images were captured and saved for further analysis.
10. Further Analysis: Immunofluorescence microscopy images were analyzed using Tissue FAXS Viewer (version 7.1.6245.141).

### 2.10 Quantification and statistical analysis

The data mining and graph presentation process, including bubble plot, KEGG, were all performed by Dr. Tom, a customized data mining system within the BGI.A p-value or adjusted p-value (FDR/Q value) <0.05 was considered statistically significant.

## 3 Results

### 3.1 Construction of a tissue block-resolved transcriptomic atlas of the developing human fetal brainstem

**FIGURE 1:**
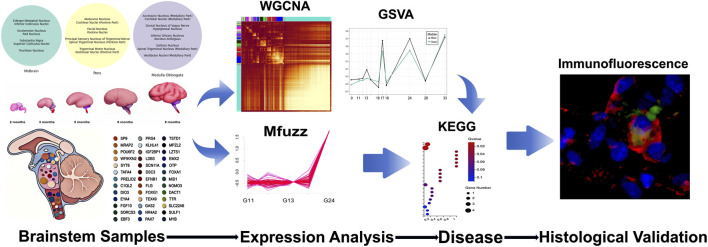
Pipeline and Schematic diagram of gestational ages of the samples. The pipeline illustrates the complete experimental workflow from sample collection, RNA sequencing and analysis, and screening for disease-associated genes, to validation of downstream protein via immunofluorescence staining.

We established the first tissue block-resolved transcriptomic atlas of the developing human fetal brainstem, spanning gestational weeks 9–33 (GW9-33). To achieve this unprecedented resolution, we collected fetal brainstem tissues of various gestational ages from the Brain Bank of Shandong University. The tissues were then cryopreserved and underwent rigorous quality control. Tissue samples were systematically harvested from anatomically distinct subregions (midbrain, pons, medulla oblongata) of the brainstem, enabling precise spatial mapping. A total of 107 distinct tissue blocks from 18 fetal specimens were analyzed (Supplementary Tables S1, S2; Figure 1; Supplementary Figures S1–S3), providing an unprecedented level of anatomical granularity.

**FIGURE 2:**
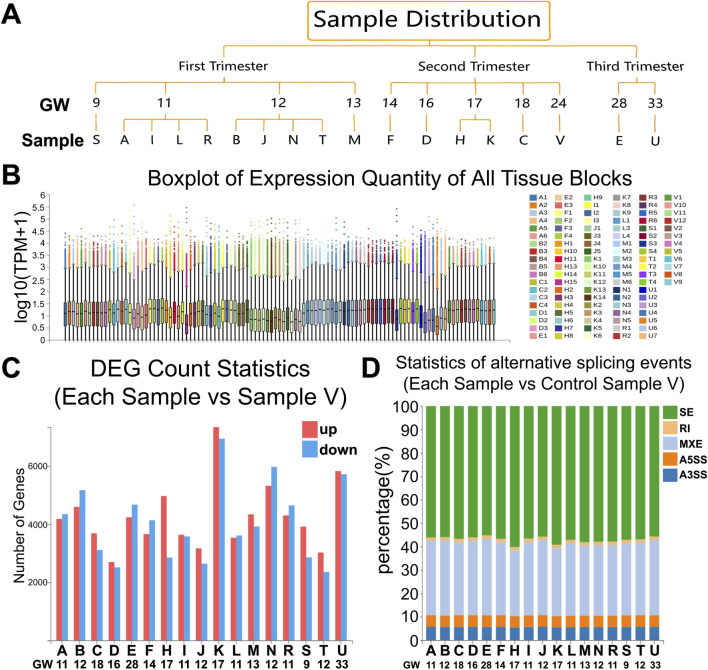
The results of RNA-seq of tissue blocks. **(A)** Dendrogram of gestational age distribution for all samples. **(B)** Distribution of gene expression quantities of all tissue blocks shown as a boxplot.Different tissue blocks are represented by different colors. **(C)** Bar plot displaying counts of differentially expressed genes (DEGs) across comparison groups (all samples compared to sample V). **(D)** Alternative splicing events across each sample (compared to sample V) Statistics include Pearson correlation coefficient (PCC) values with PV = 16.37% and SD = 4.185.Different colors represent distinct types of alternative splicing: Skipped Exon (SE), Alternative 5′Splice Site (A5SS), Alternative 3′Splice Site (A3SS), Mutually Exclusive Exons (MXE), and Retained Intron (RI).

RNA sequencing (RNA-seq) was performed on these spatially partitioned samples, generating comprehensive gene expression profiles across multiple critical developmental stages (Figures 2A,B). Differential gene expression analysis, using the 24-week samples as a reference, identified a large number of dynamically regulated genes across gestational weeks (Figure 2C), with the most substantial differences (over 14,000 genes) observed at 17 weeks, highlighting a major transcriptional shift during mid-gestation. Different types of alternative splicing events exhibited similar proportions across all samples (Figure 2D).

A comparative analysis of RNA-seq data from the fetal brainstem (across multiple gestational weeks) and adult brain tissue (H0351.2001) revealed a distinct transcriptomic shift (http://human.brain-map.org/static/download) (Shen et al., 2012). Differential expression analysis, coupled with GO and KEGG enrichment (Supplementary Figure S4), demonstrated marked activation of genes and pathways related to cell proliferation and neurogenesis in the fetal brainstem. In contrast, the adult brain tissue showed predominant expression of genes associated with synaptic function and immune regulation. This systematic comparison elucidates the molecular transition from development to maintenance during maturation.

This foundational dataset empowers researchers to systematically query the expression levels of specific genes across distinct anatomical subregions at various developmental time points (gestational weeks) during fetal brainstem development. Importantly, to ensure broad utility and advance collaborative research in neurodevelopment, all datasets—including raw sequencing data, processed expression matrices, and analytical code—will be published online. This open-access resource provides an indispensable resource for deciphering the molecular underpinnings of human brainstem development and its links to neurological disorders.

### 3.2 Analysis of gene expression during fetal brainstem development

#### 3.2.1 WGCNA

**FIGURE 3:**
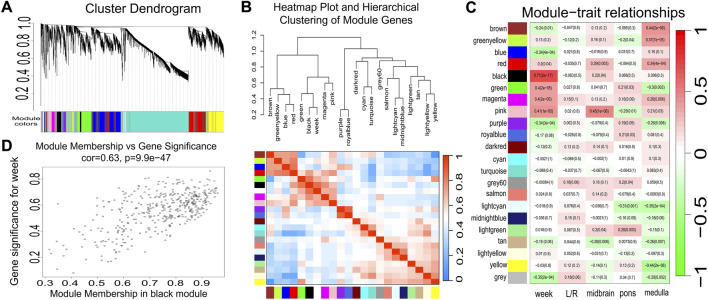
The results of WGCNA of gene expression during fetal brainstem development. **(A)** Clustering dendrogram of genes, with dissimilarity based on topological overlay with designed module colors. **(B)** heatmap plot and hierarchical clustering of module hub genes that summarize the modules yielded in the clustering analysis. Gestational week shows the strongest correlation with the Black module in hierarchical clustering. **(C)** Module-trait associations.Each row corresponds to a module eigengene, column to a trait.Each cell contains the corresponding correlation and p-value. **(D)** A scatterplot showing the highly significant correlation between Gene Significance (GS) for gestational week and Module Membership (MM) in the black module.

We identified a comprehensive set of genes expressed during fetal brainstem development. Using Weighted Gene Co-expression Network Analysis (WGCNA), cluster analysis of all detected genes was performed to characterize gene expression patterns. WGCNA is a systems biology approach designed to describe gene association patterns across different samples. By grouping genes into modules with strongly covarying expression patterns, this method identifies highly co-expressed gene modules. We incorporated gestational age, lateral position, and relative anatomical location of each tissue block as key traits in the analysis. The resulting network construction yielded 22 distinct gene modules, each demonstrating unique functional characteristics. The clustering dendrogram of genes, color-coded by module membership, revealed groups of highly co-expressed genes (Figure 3A). Notable variation was observed in the correlations among different modules, with the Black module exhibiting the strongest correlation with gestational age (Figure 3B). Module-trait relationship analysis further identified significant correlations between specific modules and biological traits, including gestational age and spatial location (Figure 3C). For example, the black module exhibited a strong correlation between module membership and gene significance for gestational age (Figure 3D), underscoring its relevance to developmental progression. Visualization of the topological overlap matrix (TOM) illustrated the overall gene network structure (Supplementary Figure S5). Genes within each module demonstrated high functional synergy, contributing to specific biological processes. Beyond high co-expression, genes within individual modules also demonstrate significant functional coherence. For instance, the turquoise module is significantly enriched with genes (such as EIF4A2, EIF4E, and EIF4G2) involved in ribosomal structure and cytoplasmic translation, indicating its central role in maintaining basal protein synthesis throughout gestation. This module already shows high activity in early pregnancy. The black module (containing ACOT8,FRAT1, KCNK1,KCNN3,KCNQ3,ABCC9,etc.) is highly correlated with gestational age. Its constituent genes are highly interconnected in function, collectively participating in processes such as neurotransmitter transport and regulation of inwardly rectifying potassium channels. The expression levels of this module increase significantly during mid-to-late gestation, suggesting its temporal importance in developmental patterning and maturation of neural electrical activity. Furthermore, the green module (including CYP1A1, CYP2C8,CYP19A1, CYP27A1, ARF6, RAB11FIP3, etc.) shows functional coordination in steroid hydroxylase activity and GTPase binding, while the blue module (e.g., CHRM4, CHRNA3, IL1R1, IL4R, IL7R, IGLL1, etc.) is enriched in functions related to transmembrane signaling receptors and immunoglobulin production. These observations reflect the functional coupling of genes within each module in regulating developmental processes. The WGCNA results highlight the complexity of functional regulation during brainstem development. Together, these results provide a comprehensive view of the co-expression landscape and functional organization of the developing fetal brainstem. Detailed information including the number of genes and primary functions of each gene module is provided in Supplementary Table S3.

Among the 22 gene modules, the black module was identified as demonstrating a strong positive correlation with gestational age, suggesting that genes associated with calcium channel activity and transmembrane transport functions are closely linked to developmental progression. For example, the *ABCC9* gene identified in the black module encodes ATP-binding cassette subfamily C member 9, a regulator of ATP-sensitive potassium channels that indirectly influences calcium signaling pathways (Zingman et al., 2007). *CALHM1* encodes calcium homeostasis modulator 1, a calcium channel critical for regulating intracellular calcium homeostasis (Dreses-Werringloer et al., 2008). Among the co-expression modules analyzed, the turquoise module contained the highest number of genes (n = 4,853) and showed enrichment for ribosomal functions. Notably, highly expressed genes in this module, including *MRPL42* and *RPS23* (Yoo et al., 2005), suggest that ribosomal biogenesis plays an essential role in fetal brainstem development. The blue module showed significant enrichment for transmembrane signaling receptor activity, containing key neural receptors including *CHRM4* and *CHRNA3* (Pancani et al., 2014). These genes are involved in regulating intercellular communication during brainstem development.

The gene modules exhibited inter-modular correlations, as shown in the WGCNA results, which display a network of relationships among the clustered modules. A strong correlation between the black and green modules was notably observed (Figure 3B). The genes within the black module are primarily associated with functions including the negative regulation of protein autoubiquitination, histamine secretion, neurotransmitter transmembrane transporter activity, and inward rectifier potassium channel activity. The genes in the green module are involved in functions such as steroid hydroxylase activity and GTPase binding, suggesting a potential intrinsic functional link between the two modules during brainstem development. Previous studies have demonstrated a close relationship between neurotransmitter transmembrane transporter activity and GTPase binding (Möhle et al., 2001). Therefore, it is plausible to hypothesize that other functions associated with these two modules may also exhibit direct or indirect interactions. Cross-module analysis further revealed a strong correlation between the blue and red modules. The blue module is primarily associated with functions including transmembrane signaling receptor activity, immunoglobulin production, and serotonin receptor signaling pathways. For example, GPR45 is associated with G protein-coupled receptor signaling (Lin and Civelli, 2004), while *HTR7* is linked to the serotonin receptor signaling pathway (Cortes-Altamirano et al., 2018). The red module is primarily associated with functions including T cell activation, peptidyl-tyrosine autophosphorylation, and histone lysine methylation. Previous studies have demonstrated a strong correlation between immunoglobulin production and T-cell activation (Grewal and Flavell, 1998). Therefore, the association between the blue and red modules suggests that other functions within these modules may also exhibit significant interrelationships. The purple and royalblue modules exhibited a strong correlation. Functional analysis revealed that the royalblue module is predominantly involved in protein glycosylation, glycoprotein biosynthesis, and metabolic regulation, while the purple module is enriched for activities related to oxidative DNA demethylation, protein tyrosine dephosphorylation, phosphoprotein dephosphorylation, and phosphatidylinositol-3-phosphate dephosphorylation. Previous studies have demonstrated that glycosylation modifications may indirectly influence DNA methylation status through the regulation of chromatin structure or related enzymatic activities (Chu et al., 2014). This evidence suggests that protein glycosylation processes may similarly modulate DNA methylation patterns during fetal brainstem development. The gene modules and interaction networks constructed via WGCNA systematically delineate the coordinated regulatory architecture and modular interaction mechanisms underlying gene functional synergy during fetal brainstem development. These findings collectively suggest that brainstem development is governed not by linear regulation of isolated pathways, but rather by multi-layered cooperative regulatory logic characterized by temporal coupling signatures and high functional synergy among modules, reflecting complex interaction patterns across spatiotemporal dimensions.

#### 3.2.2 GSVA

**FIGURE 4:**
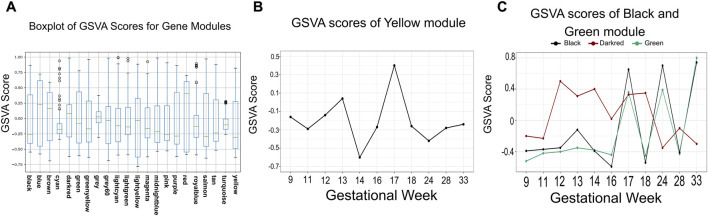
The results of GSVA of gene expression during fetal brainstem development. **(A)** Boxplot of GSVA Scores for Gene Modules. **(B)** Changes in the GSVA score of black, green and darkred module across gestational week. **(C)** GSVA score of the yellow module across gestational week.

Because individual gene expression varies substantially, solely using expression levels is inadequate for assessing gene module function. Gene Set Variation Analysis (GSVA) was employed to interrogate the 22 WGCNA-derived gene modules, systematically assessing their activity dynamics and functional roles during development (Figure 4A). The resulting scores quantitatively represent the activity levels of each gene module. The GSVA scores are provided in Supplementary Table S4. (The GSVA scores range from −1 to 1, with values approaching 1 indicating higher module activity and values approaching −1 representing greater suppression).

The scoring results revealed that the expression levels were not directly correlated with the activity of gene modules. For example, the GSVA analysis revealed a notable discrepancy between gene expression levels and module activity. Despite a significant upregulation of expression levels for most modules, certain modules exhibited decreased GSVA scores at the 17th gestational week (Supplementary Figure S6). This may indicate a potential narrowing of functional divergence among distinct gene modules in the fetal brainstem during advanced gestational stages.

For each individual module, the GSVA scores exhibited distinct temporal dynamics. For instance, in the yellow module, while the expression levels increase rapidly with gestational age, the GSVA score peaks at 17 weeks (Figure 4B). After the 17th gestational week, expression levels were significantly upregulated, whereas GSVA scores markedly decreased, suggesting that genes and functions associated with the yellow module (including inorganic phosphate transmembrane transporter activity and α-glucosidase activity) were more active in brainstem development at this stage but declined during late pregnancy. Furthermore, consistent with their co-expression relationship in the WGCNA, the green and black modules also showed a significant correlation in GSVA scores (Figure 4C), providing additional evidence for their coordinated activity.

### 3.3 Temporal paradigm induction of gene expression

**FIGURE 5:**
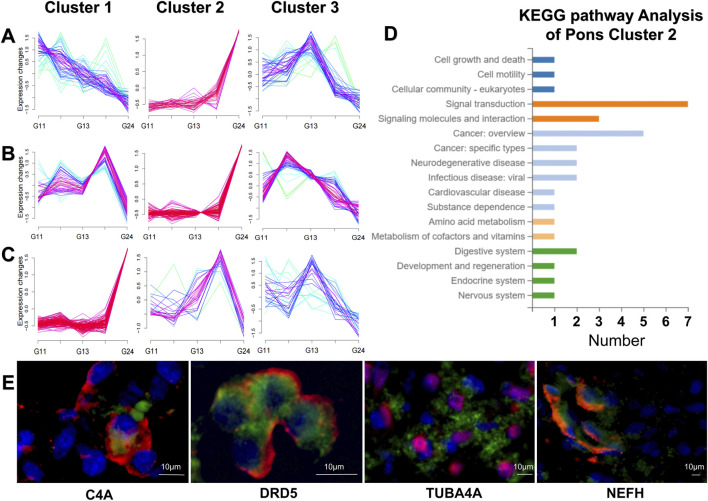
The results of Mfuzz, KEGG and Immunofluorescence images. **(A)** Line graph of gene expression dynamics of cluster 1,2,3 of midbrain. **(B)** Line graph of gene expression dynamics of cluster 1,2,3 of pons. **(C)** Line graph of gene expression dynamics of cluster 1,2,3 of medulla. **(D)** Bar graph of KEGG pathway analysis of cluster 2 of pons. **(E)** Immunofluorescence images of horizontal sections of the fetal pons at 24 gestational weeks, showing the downstream proteins of C4A, DRD5, TUBA4A, and NEFH. The target proteins are shown in green, nuclei were stained with DAPI (blue), and neurons were labeled with neuron-specific markers (red). MAP2 was used for TUBA4A, while NeuN was used for C4A, DRD5, and NEFH.

To characterize temporal expression dynamics, we analyzed fetal brainstem samples collected at gestational weeks (GW) 11, 12, 13, 17, and 24. These time points were chosen due to their comprehensive coverage of key gestational windows and the presence of sufficient sampling sites across all samples. Adjustment of LogFC values in Mfuzz enabled identification of midbrain, pons, and medulla oblongata genes that exhibited significant temporal expression dynamics across gestational weeks. These genes were clustered based on their distinct temporal expression patterns (i.e., temporal paradigms), resulting in three gene clusters for each anatomical structure. Genes within each cluster shared similar temporal expression paradigms (Figures 5A–C), with the complete gene lists for these clusters provided in Supplementary Table S5.

Quantitative trajectory analysis revealed extremely high expression in Cluster 1 of the midbrain tri-cluster system during early pregnancy, which decayed exponentially in subsequent gestation.Genes in Cluster 2 exhibited a gradual increase in expression levels throughout gestation, followed by a sharp rise at 24 gestational weeks. In contrast, genes in Cluster 3 showed an upward trend prior to 13 gestational weeks, peaked at 13 gestational weeks, and subsequently declined. Among the three pontine clusters, genes in Cluster 1 reached peak expression at 17 gestational weeks, followed by a subsequent decline. Cluster 2 genes maintained stable expression levels during early-to-mid gestation but showed a marked increase during late gestation (at 24 gestational weeks). In contrast, Cluster 3 genes attained maximal expression at 13 gestational weeks, with subsequent gradual decline. Among the three medullary clusters, genes in Cluster 1 maintained stable expression levels during early-to-mid gestation but exhibited a marked increase during late gestation (at 24 gestational weeks). Cluster 2 genes peaked in expression at 17 gestational weeks, followed by a significant decline subsequently. In contrast, Cluster 3 genes reached maximal expression at 13 gestational weeks, with subsequent gradual decline.

Similarities in temporal patterns were observed among gene clusters identified in the midbrain, pons, and medulla oblongata. For instance, midbrain Cluster 2, pons Cluster 2, and medullary Cluster 1 exhibited nearly identical temporal patterns. Similarly, medullary Cluster 3 and midbrain Cluster 3 showed analogous temporal dynamics. These findings suggest similar temporal regulatory mechanisms among these gene groups during specific gestational weeks.

Through the analysis above, a comprehensive gene expression profile of fetal brainstem development was constructed, facilitating the identification and query of specific genes involved in this developmental process. For example, to examine the expression pattern of the CHAT gene in the fetal pons, we first used Mfuzz results to identify that CHAT belongs to Cluster 2 of the pons. As shown in Figure 5B, its temporal expression pattern exhibits higher levels during the late gestational period. Additionally, WGCNA results revealed that this gene belongs to the turquoise module, which is predominantly associated with ribosomal functions, suggesting a close link between CHAT (acetylcholine synthesis-related gene) (Luo et al., 2024) and ribosome-related processes during brainstem development. The functional activity (Supplementary Table S4) of this module was significantly elevated in the medulla oblongata at 17 weeks of gestation (GSVA). Using the above analysis, we can efficiently query the expression levels, activity patterns, and temporal dynamics of specific genes in any region of the fetal brainstem throughout gestation. This information provides a foundation for further studies on disease associations and functional roles of these genes.

### 3.4 Identification of genes associated with neurological disorders

Analysis of temporal clusters revealed that pontine cluster 2 was particularly enriched for genes associated with the nervous system and neurodegenerative diseases (Figure 5D). This cluster was selected for further screening. Since this study employed fetal brainstem samples, the investigation focused on neurological disorders with significant congenital contributions, particularly amyotrophic lateral sclerosis (ALS), attention deficit hyperactivity disorder (ADHD) and schizophrenia. Notably, many of the diseases under investigation lack definitive histopathological markers—especially at early stages—and are instead characterized by clinical biomarkers. This underscores the importance of probing their potential developmental origins through gene expression patterns in fetal brainstem regions. Among these regions, the pons is a key brainstem structure which serves as an integrative hub for autonomic, sensorimotor, and neuromodulatory functions. It may undergo early developmental alterations that predispose individuals to later neuropsychiatric illness. Table 1 lists the genes from pontine cluster 2 that are associated with these diseases and summarizes disease-associated genes identified through screening, their assigned gene modules, anatomical regions with specific temporal patterns, and associated neuropsychiatric disorders. To facilitate further validation, these genes were selected for downstream analysis of their protein expression profiles.

**TABLE 1:** Neuropsychiatric disorder-associated genes.

Gene	Module	Anatomical parts	Disease
*C4A*	Black	Pons, Midbrain	Schizophrenia
*DRD5*	Yellow	Pons	ADHD
*NEFH*	Brown	Pons	ALS
*TUBA4A*	Salmon	Pons	ALS

This table summarizing disease-associated genes identified through screening, their assigned gene modules, anatomical regions with specific temporal patterns, and associated neuropsychiatric disorders.

Among these genes, *C4A* encodes the acidic form of complement component 4, which is part of the classical activation pathway (Gomez-Arboledas et al., 2021). *DRD5* encodes the D5 subtype of dopamine receptor, a G protein-coupled receptor that stimulates adenylate cyclase. Previous studies have indicated that this receptor is predominantly expressed in neurons within the brain’s limbic regions (Beaulieu and Gainetdinov, 2011). *NEFH* encodes the heavy neurofilament protein, which is commonly used as a biomarker for neuronal injury (Runge et al., 2023). Mutations in this gene are associated with susceptibility to amyotrophic lateral sclerosis (ALS). The *TUBA4A* gene encodes α-tubulin, which forms heterodimers with β-tubulin to constitute the cytoskeleton. Previous studies indicated that *TUBA4A* is also associated with ALS (Smith et al., 2014).

By comparing the gene expression data in the midbrain and medulla oblongata, distinct expression patterns of these genes were observed not only in the pons but also in other brainstem regions. For instance, pronounced temporal patterns of *C4A* are observed in both the pons and the midbrain (Table 1). This suggests the existence of a spatiotemporally specific gene regulatory network across brainstem subregions during development. Such spatiotemporally coordinated expression dynamics may underlie the programmed regulatory mechanisms of neural system development and provide critical temporal clues for elucidating the pathogenesis of related neurodevelopmental disorders. Prenatal testing of the disease-associated genes identified in this study could potentially enable early screening and monitoring of abnormalities during brainstem development, thereby allowing for interventions that may reduce the incidence of associated neurological disorders.

### 3.5 Immunofluorescence staining validation of downstream protein expression for neurological disorder-associated genes

We next validated the expression of proteins encoded by the screened disease-associated genes (Table 1) using immunofluorescence in the fetal brainstem. Given the superior sequencing coverage in 24-week specimens, a fetal brainstem sample of this gestational age was acquired from the Brain Bank of Shandong University for comparative analysis. Immunofluorescence staining was performed on frozen sections from different regions to detect the downstream proteins of the screened genes (Figure 5E; Supplementary Figure S7; Supplementary Figure S8).

We prioritized the *NEFH* gene for initial validation. To examine the expression of the *NEFH*-encoded protein in the pontine region, immunofluorescence staining was performed on horizontal sections of the pons obtained from a 24-week-old fetal brain, with the results presented in Figure 5E. The distinct color contrast between the green fluorescence (*NEFH*-encoded heavy neurofilament proteins) and blue DAPI nuclear staining clearly demonstrated the cytoplasmic distribution of neurofilament proteins as integral cytoskeletal components. Notably, *NEFH* was clustered within the Brown module in our WGCNA co-expression network. This module demonstrated a mean GSVA score of 0.47 across pontine subregions at GW24, statistically corroborating the high immunohistochemical expression. These findings suggest that the protein encoded by this gene exhibits high expression levels and significant functional potency during development. The alterations in *NEFH* expression or function may disrupt brainstem homeostasis and neural circuitry integrity, potentially contributing to the pathogenesis of amyotrophic lateral sclerosis (ALS) and related neurological disorders.

To examine the expression of *C4A*-encoded complement components in the pontine region, we performed double-label immunofluorescence on horizontal sections of the pons from the same 24-week-old fetal brain. The results are shown in Figure 5E. Complement *C4A* protein was visualized using green fluorescence, and spatial co-localization with the DAPI nuclear counterstain was observed. The results revealed primary localization of the protein in neuronal plasma membrane regions. Multi-regional analysis revealed increased *C4A* expression in the pons, a finding consistent with the Black module identified by WGCNA. This module demonstrated an average expression level of 132.45(FPKM) across pontine subregions and a mean GSVA score of 0.41, findings that were corroborated by immunofluorescence analysis. Previous studies have demonstrated significant correlations between the gene dosage effects of complement C4 and the neurodegenerative progression in schizophrenia (Jenkins et al., 2023).

Subsequent validation confirmed expression of the *DRD5* gene product. The traditional view holds that *DRD5* is primarily expressed in the limbic system (e.g., hippocampus and nucleus accumbens) and is involved in emotional regulation (Meador-Woodruff et al., 1992). Immunofluorescence co-localization assays were employed to validate protein expression of the *DRD5*-encoded dopamine receptor in the aforementioned 24-week fetal horizontal pontine sections. The results are comprehensively documented in Figure 5E. Green fluorescence labeling of the *DRD5*-encoded receptor protein demonstrated predominant localization to neuronal plasma membrane regions. Multi-planar sectional analysis revealed elevated expression of the *DRD5*-encoded protein within pontine regions, demonstrating spatial congruence with its transcriptional profile across pontine subregions. This expression pattern paralleled the GSVA score characteristics (mean = 0.25) of its associated Yellow module in WGCNA analysis, indicating robust concordance between molecular and protein-level observations. Previous studies have confirmed a strong association between genetic polymorphisms in the *DRD5* gene and synaptic plasticity impairments in schizophrenia (Zhao et al., 2014). Collectively, our findings suggest that *DRD5* dysfunction during late-stage fetal pontine development may disrupt dopaminergic signaling. This disruption could induce a dysregulation of developmental timing in the brainstem, which may represent an early pathological process contributing to schizophrenia.

As delineated in preceding sections, both *NEFH* and *TUBA4A* demonstrate robust associations with amyotrophic lateral sclerosis (ALS) (Al-Chalabi et al., 1999). The *NEFH* gene encodes the neurofilament heavy chain protein, a critical subunit of neurofilaments that constitute the axonal cytoskeleton essential for maintaining neuronal structural integrity (Yuan and Nixon, 2021). Similarly, the *TUBA4A* gene encodes alpha-tubulin, a core component of microtubule networks that form the dynamic scaffolding system within the neuronal cytoskeleton (Ganne et al., 2023). Our fluorescence imaging results further reveal significant expression of both *NEFH* and *TUBA4A* genes in the fetal pons (Figure 5E). This spatiotemporal expression pattern is relevant given that previous studies have established that *TUBA4A* mutations disrupt cytoskeletal dynamics, constituting a pathogenic mechanism in ALS (Smith et al., 2014). Notably, structural abnormalities of the *NEFH*-encoded neurofilament heavy chain, a core component of the neuronal axonal cytoskeleton, have also been implicated in ALS pathogenesis (Guo et al., 2024). Collectively, these findings provide critical insights for developing prenatal diagnostic biomarkers and early screening protocols targeting cytoskeletal pathophysiology in neurodevelopmental disorders.

## 4 Discussion

This study presents the first tissue block-resolved transcriptomic atlas of the developing human brainstem, spanning early gestation to late gestation (9–33 weeks). By integrating RNA-seq data from anatomically precise tissue blocks with systems biology approaches (WGCNA, GSVA, Mfuzz) and immunofluorescence validation, we provide a foundational resource that delineates the molecular programs underlying fetal brainstem development. Our key findings reveal dynamic, stage-specific gene co-expression networks, identify genes with strong links to neurological disorders, and demonstrate their temporal expression patterns. This work moves beyond prior bulk-tissue studies by offering regional and temporal resolution, thereby illuminating the developmental origins of brainstem-mediated functions and associated pathologies.

In this study, our tissue block-resolved RNA-seq strategy provides a perspective that is complementary to the widely used single-cell transcriptomic profiling of telencephalic regions, with a regional focus (Braun et al., 2023). While single-cell methods excel at revealing cellular heterogeneity, we leveraged precise microdissection of the brainstem and regional bulk RNA-seq to successfully obtain an overview of region-specific gene expression within this complex structure. This work thereby addresses a critical gap in transcriptomic data on human fetal brainstem development.

### 4.1 Decoding brainstem development through gene co-expression networks

Using WGCNA, we successfully simplified the complex transcriptomic data into 22 manageable and biologically meaningful co-expression modules. This systems-level view reveals that brainstem development is not governed by isolated genes but by coordinated networks functioning in concert. Several modules showed strong correlations with gestational age, highlighting the temporal specificity of developmental processes.

For instance, the turquoise module (enriched for ribosomal biogenesis and translation) was highly active throughout gestation (Yoo et al., 2005). This underscores the immense biosynthetic demand underlying the development of the complex neural architectures of the brainstem. Conversely, the black module (enriched for calcium channel activity and transmembrane transport) became increasingly active with advancing gestational age (Zingman et al., 2007; Dreses-Werringloer et al., 2008). This suggests a shift towards the functional maturation of neurons, particularly in electrophysiological properties and synaptic communication, which is consistent with findings in other brain regions (Johnson et al., 2009; Zhong et al., 2020).

Furthermore, the strong correlations between specific modules (e.g., black-green and blue-red) suggest coordinated regulation between distinct biological processes. For example, the link between the blue module (transmembrane signaling receptor activity) and the red module (T-cell activation and phosphorylation) may point to an intricate, developmentally programmed interaction between neural and immune functions in the developing brainstem (Grewal and Flavell, 1998).

### 4.2 Temporal and spatial dynamics of gene expression

A major advantage of our study is its capacity to track gene expression over time and across different brainstem subregions (midbrain, pons, and medulla). Our Mfuzz analysis classified genes into distinct temporal clusters, each representing a unique expression trajectory.

Crucially, we discovered that similar temporal patterns recurred across different anatomical regions. For example, a pattern of late-gestational upregulation occurred in midbrain cluster 2, pons cluster 2, and medullary cluster 1. This convergence implies that shared regulatory mechanisms may govern certain developmental programs (such as late-stage neuronal maturation or circuit formation) across the entire brainstem, despite its anatomical diversity. This finding provides a novel perspective on the coordinated development of the brainstem (Kostović and Judas, 2010).

### 4.3 Linking developmental expression to neurological disorders

One of our key findings is the experimental validation of the expression of downstream proteins encoded by neurological disease-associated genes in fetal brainstem samples. We focused on genes within the pons that both exhibited strong temporal dynamics and were enriched for nervous system disorders.

Our data reveal that key risk genes for major disorders are highly dynamic during fetal development. For example, *C4A*, a complement component linked to synaptic pruning defects in schizophrenia (Kim et al., 2021; Seka et al., 2016), was active in the pons. The spatiotemporal specificity of these genes suggests they participate in specific developmental events, such as the maturation of motor circuits. Therefore, dysregulation of these genes during this critical fetal window could disrupt normal developmental programs, potentially establishing a hidden vulnerability that manifests as disease later in life. The spatiotemporal expression patterns of synaptic genes we observed likely underlie the process of synaptogenesis in the fetal brainstem, as reported in classical immunohistochemical studies (Sarnat et al., 2013). Our immunofluorescence validation confirmed the expression of the protein products encoded by these genes in the predicted anatomical subregions, thereby providing strong support for the transcriptomic findings and establishing a link between developmental biology and neuropathology.

Although it is methodologically challenging to link fetal brainstem development to disorders that manifest later in life, this perspective is grounded in the 'developmental origins of health and disease’ (DOHaD) paradigm (Faa et al., 2016). The core hypothesis posits that the fetal period represents a critical window for the establishment of neural circuit integrity and cellular homeostasis. Perturbations in the expression of key genes—such as those involved in cytoskeletal architecture (*NEFH*, *TUBA4A*), synaptic pruning (*C4A*), or neurotransmitter signaling (*DRD5*)—during this precise developmental program can fundamentally alter brainstem maturation. The brainstem, as a central hub for nascent motor, sensory, and modulatory pathways, is particularly vulnerable. Such early-life molecular dysregulation may not cause immediate pathology but can install a latent vulnerability or a “hidden pathology” that remains clinically silent. This vulnerability only manifests as overt disease (e.g., ALS in adulthood or schizophrenia in early adulthood) decades later, upon exposure to secondary insults, age-related physiological decline, or additional genetic and environmental factors that challenge the already compromised neural systems. Therefore, our observations do not indicate active disease in the fetus, but instead position the fetal brainstem as the primary site for the developmental programming of future neurological disease susceptibility.

### 4.4 Limitations and future directions

Although our study establishes a high-resolution transcriptomic map, its basis in bulk RNA-seq inherently limits the resolution of cellular heterogeneity. The observed signals represent an average across all cell types in a tissue block. Deconvolving these aggregated signals into distinct cell-type-specific signatures and lineage trajectories will require future studies that apply single-nucleus RNA sequencing (snRNA-seq) to comparably annotated samples (Tian et al., 2023).

Furthermore, our gestational window, while extensive, does not cover the earliest embryonic stages when initial patterning occurs. Integrating data from earlier time points would provide a more comprehensive understanding. It is important to note that the brainstem samples in this study were derived from aborted fetuses. Given that widespread prenatal screening has led to most pregnancy terminations occurring in the first trimester, the availability of specimens from later gestational stages was limited, which resulted in a non-uniform distribution of samples across the temporal axis. It will be essential for future research to incorporate a larger sample collection to overcome this constraint. Finally, establishing a causal link between the identified genes, modules, and their biological outcomes will require functional validation in model systems.

## 5 Conclusion

In summary, this study presents the first tissue block-resolved transcriptomic atlas of the developing human fetal brainstem from GW9 to 33. We identified 22 co-expression modules representing core regulatory networks involved in brainstem maturation. These modules were linked to key neurodevelopmental processes and neurological disorders, providing a public resource for exploring gene activity across brainstem regions and developmental stages. Functional analyses revealed coordinated spatiotemporal programs (including ribosomal biogenesis, calcium signaling, and synaptic function) and specifically implicated disorder-associated genes such as *NEFH*. The spatiotemporal expression patterns of these genes suggest a prenatal origin for certain disease mechanisms. The spatiotemporal expression patterns of these genes suggest a prenatal origin for certain disease mechanisms. Although limited by the resolution of bulk RNA-seq, this work establishes a foundational resource for future studies and highlights candidate mechanisms for early diagnosis and intervention.

## Data Availability

The raw and processed RNA-seq data have been deposited in the NCBI Gene Expression Omnibus (GEO) under the accession number GSE307537.
