# Supplementary material for: Tissue block-resolved developmental transcriptomic atlas of human fetal brainstem reveals gene modules with implications for neurological disorders
Source: Front Cell Dev Biol. 2025 Nov 28;13:1674967. doi: 10.3389/fcell.2025.1674967 (PMC12698620; doi:10.3389/fcell.2025.1674967)
Supplement: Supplementary file 1 [file DataSheet1.pdf]

Table S1.Sampling Gender and GW Distribution

Sample	Gender	GW
A	Male	11
B	Male	12
C	Male	18
D	Female	16
E	Male	28
F	Female	14
H	Male	17
I	Male	11
J	Male	12
K	Male	17
L	Female	11
M	Male	13
N	Female	12
R	Male	11
S	Male	9
T	Male	12
U	Male	33
V	Male	24

Table S1 presents the gender and GW distribution of all samples.

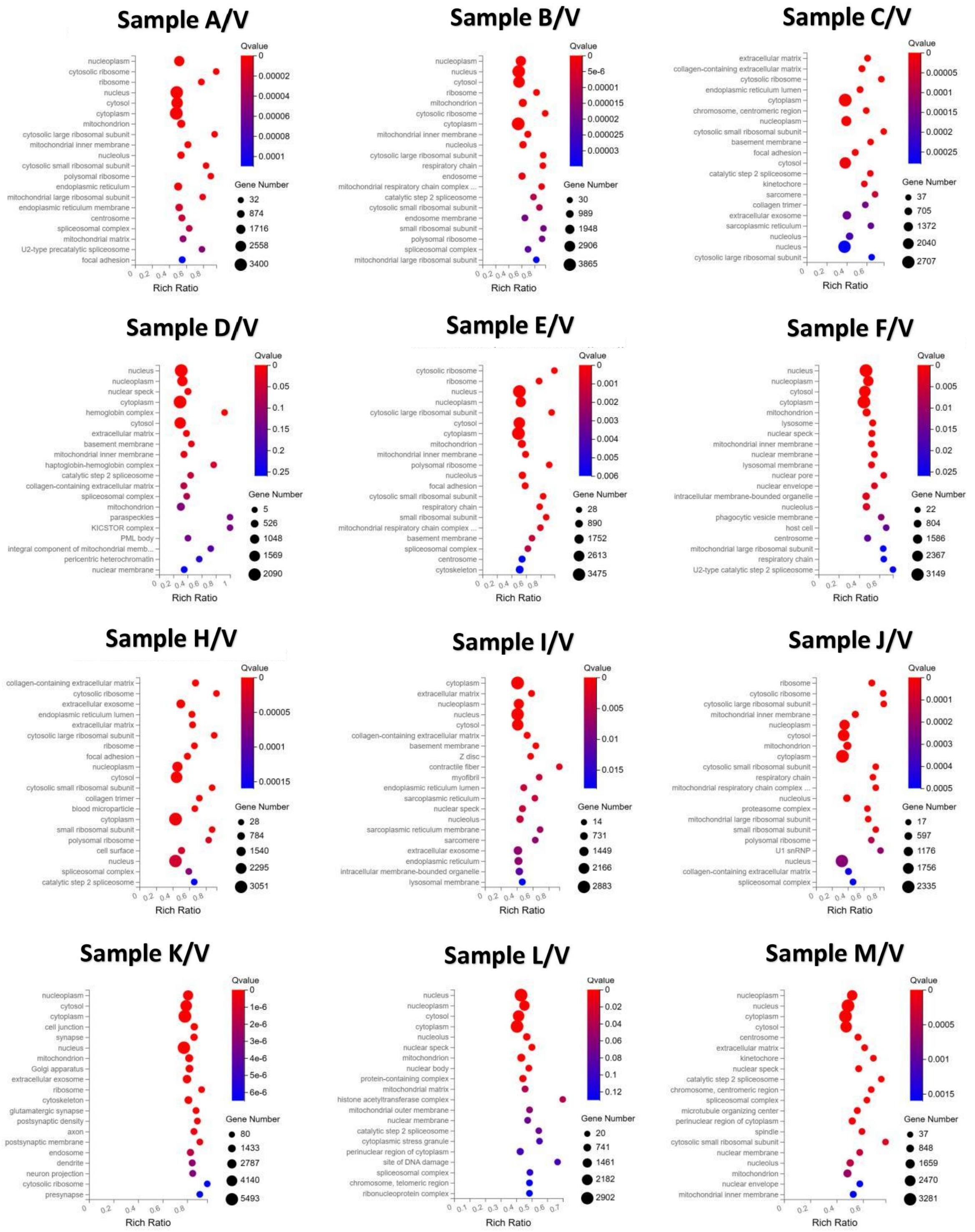

Figure S1. Bubble plots of KEGG enrichment analysis

Bubble plots of KEGG enrichment analysis of differentially expressed genes (DEGs) for each sample compared to Group V (Samples A, B, C, D, E, F, H, I, J, K, L, M versus Group V).

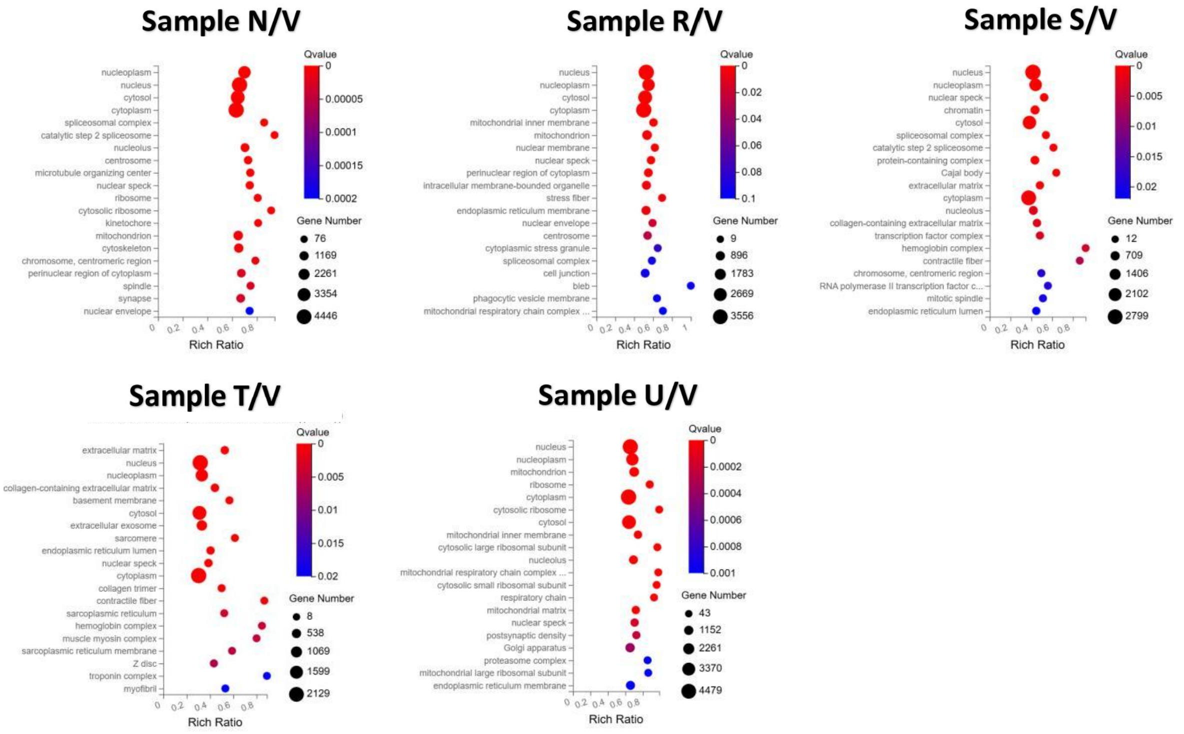

Figure S2. Bubble plots of KEGG enrichment analysis

Bubble plots of KEGG enrichment analysis of differentially expressed genes (DEGs) for each sample compared to Group V (Samples N, R, S, T, U versus Group V).

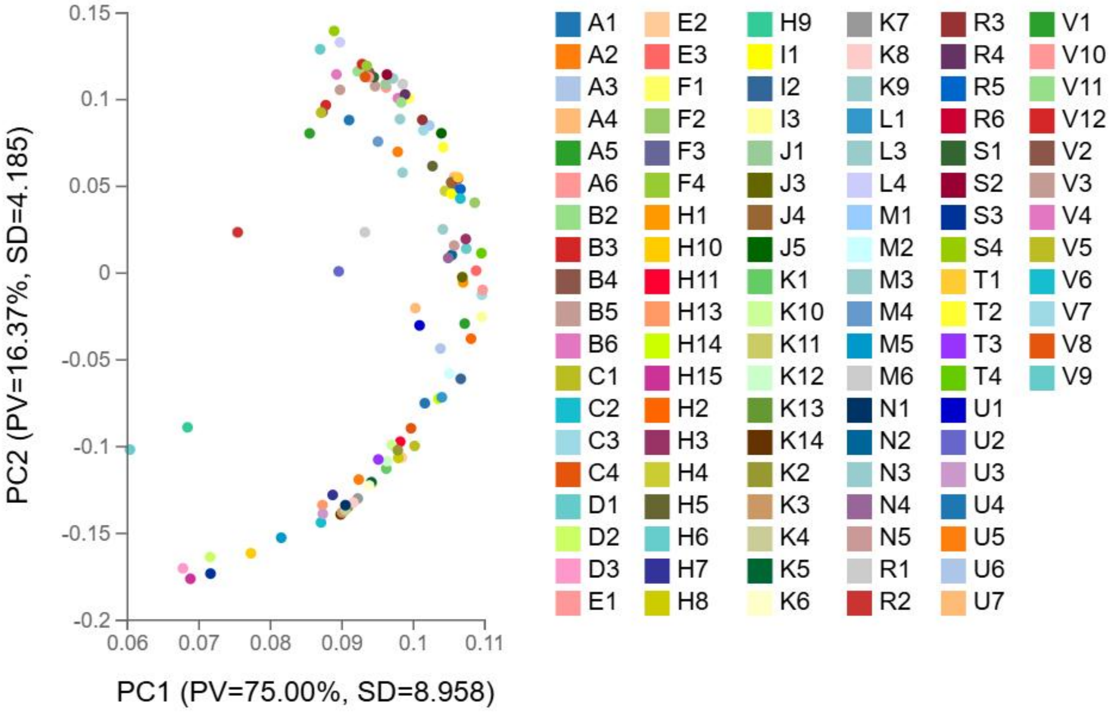

Figure S3. Principal Component Analysis of All Samples

Dimensionality reduction was performed on multiple variables, including gestational age, expression levels, anatomical location, and laterality.

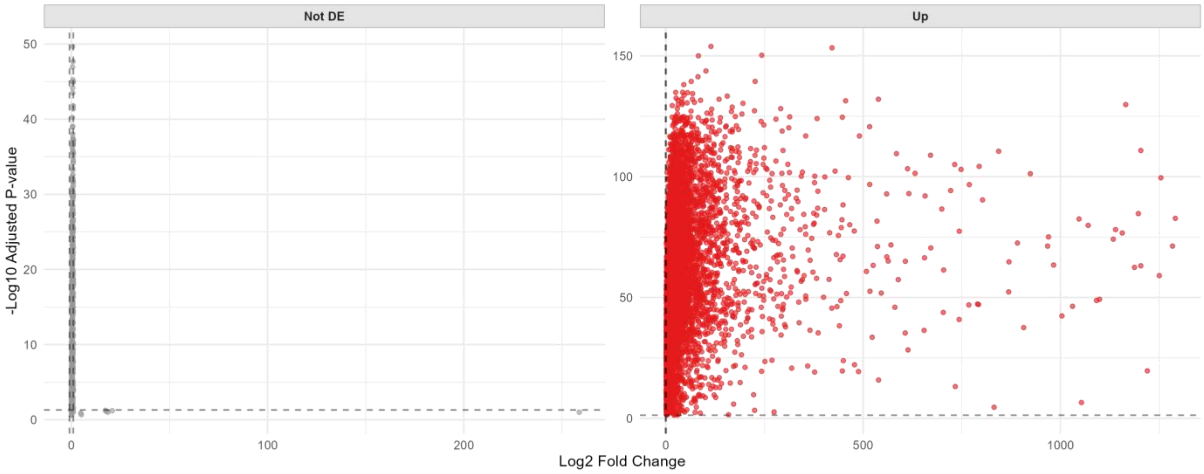

Figure S4. Volcano Plot of Differential Expression Analysis

Volcano plot grouped by expression status. The left panel ("Not DE") shows genes that are not differentially expressed, with points clustered near zero log2 fold change and low -log10 adjusted P-values. The right panel ("Up") displays genes that are upregulated, characterized by a wide range of positive log2 fold changes and high -log10 adjusted P-values (indicated by the red points). The x-axis represents Log2 Fold Change, and the y-axis represents -Log10 Adjusted P-value. Dotted lines denote the thresholds for significant differential expression.

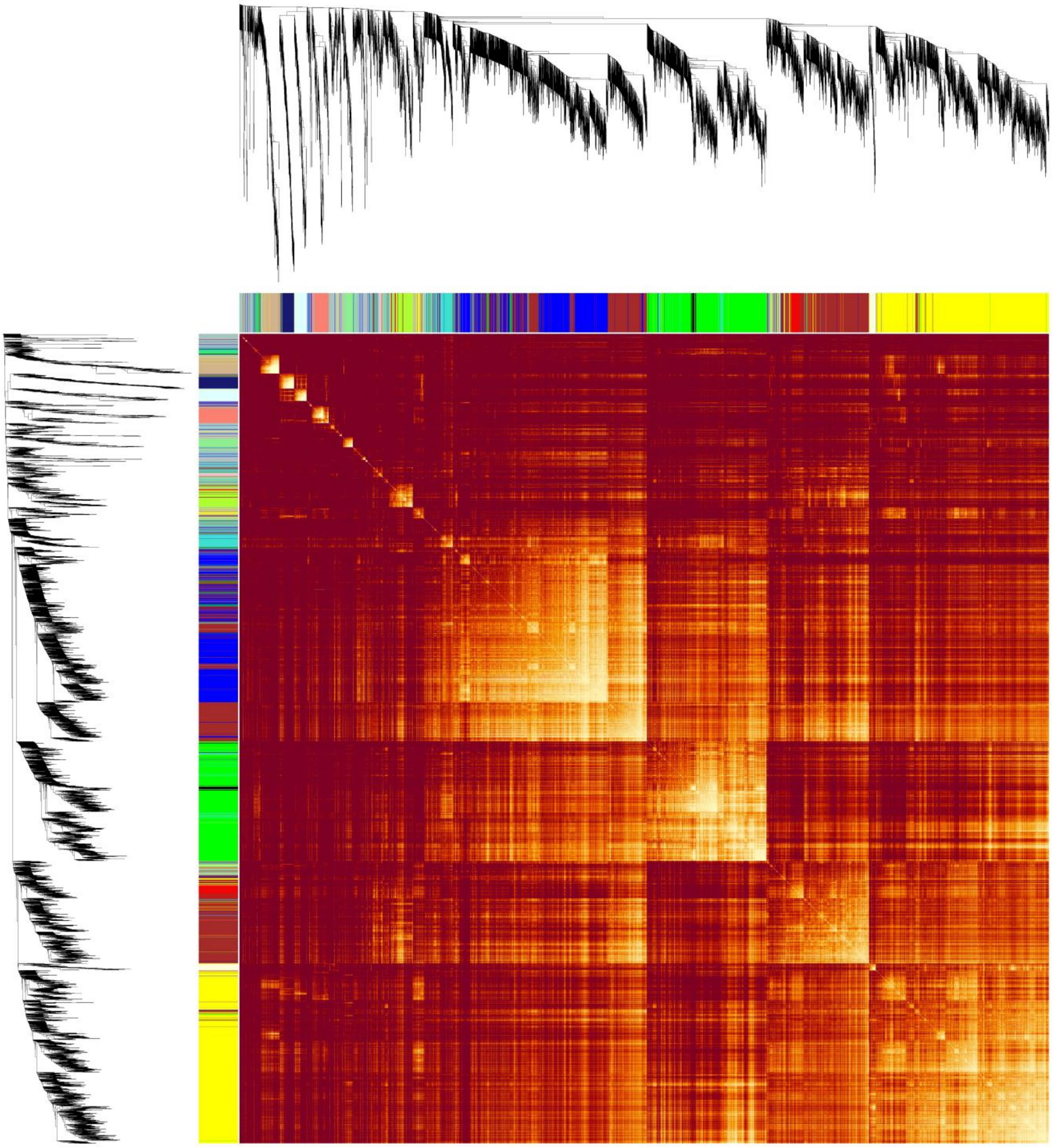

Figure S5. Gene Network Heatmap

Visualizing the gene network using a heatmap plot.The heatmap depicts the Topological Overlap Matrix(TOM) among all genes in the analysis.Light color represents low overlap and profressively darker red color represents higher overlap.Different gene modules are shown by colors along the left side and the top.

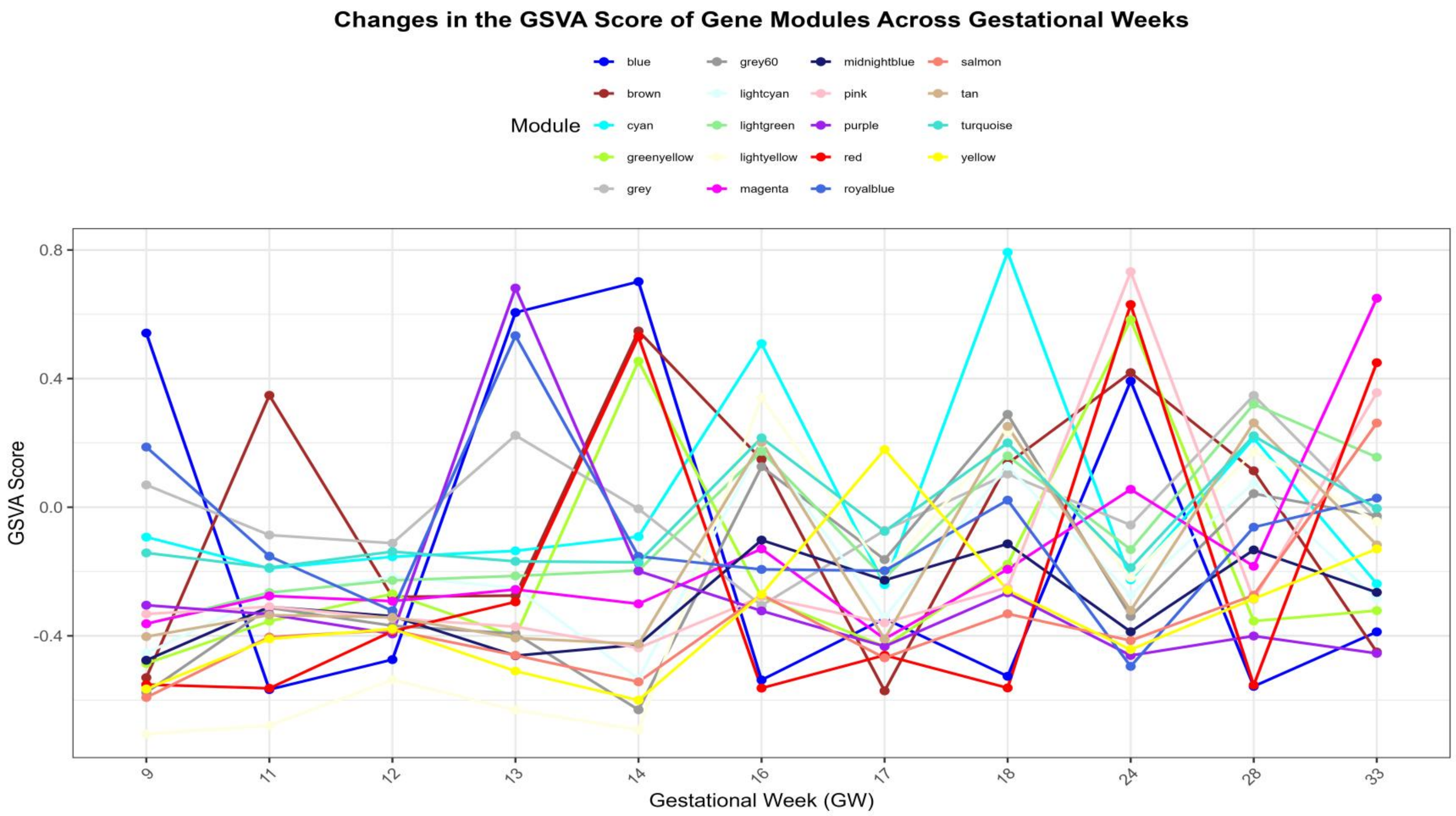

Figure S6.Changes in the GSVA Score of Gene Modules Across Gestational Weeks

The line chart depicts changes in GSVA scores of distinct gene modules across various gestational ages. The x-axis represents gestational age, while the y-axis indicates the mean GSVA score. Lines in distinct colors correspond to different modules.

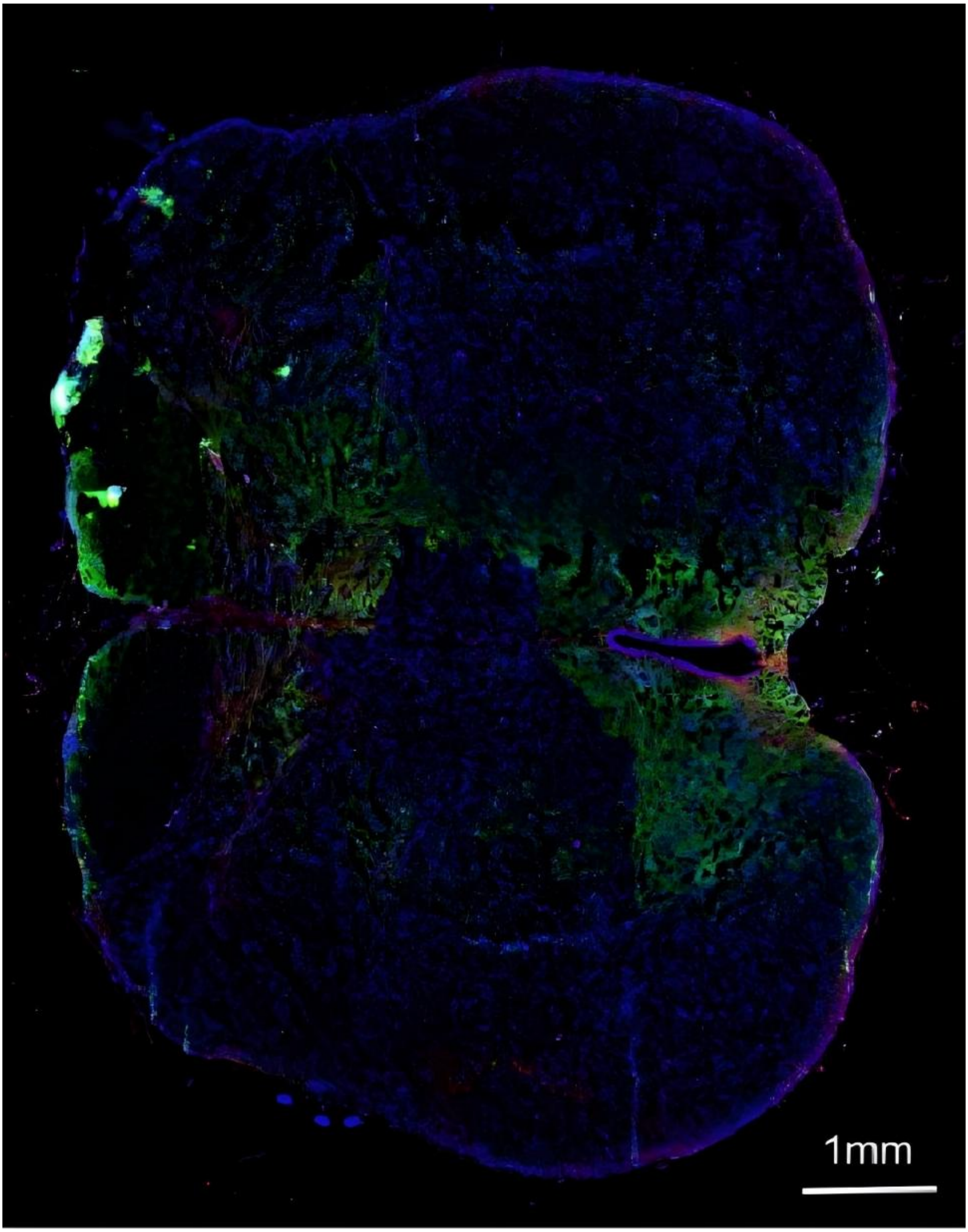

Figure S7. Immunofluorescence image of a transverse section of the fetal pons

Immunofluorescence staining of a transverse section at the pontine level from a 24-gestational-week formalin-fixed fetal brain, showing expression of downstream proteins of NEFH and TUBA4A.

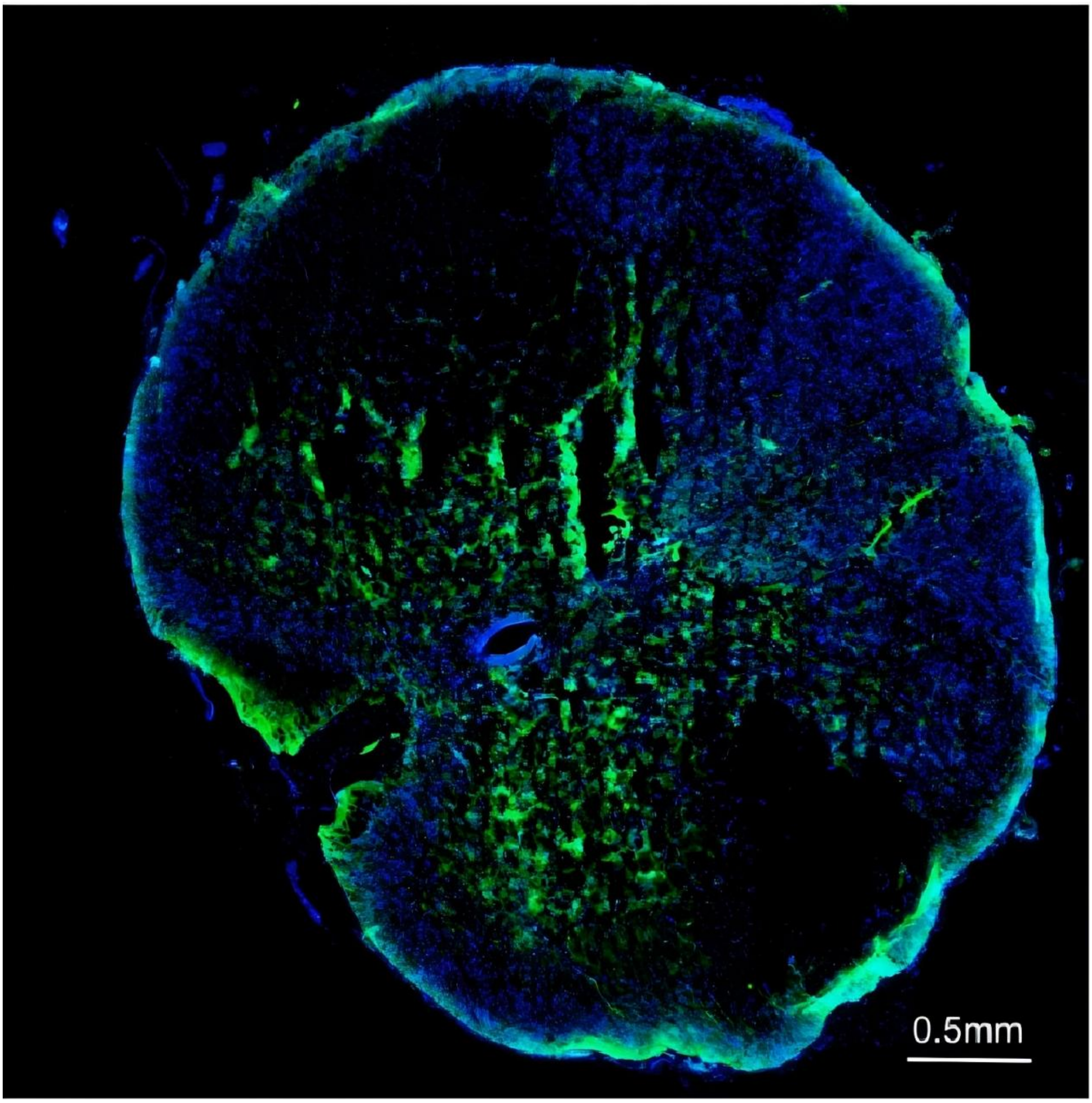

Figure S8. Immunofluorescence image of a transverse section of the fetal medulla oblongata

Immunofluorescence staining of a transverse section at the medulla oblongata level from a 24-gestational-week formalin-fixed fetal brain, showing expression of downstream proteins of TUBA4A.

Table S2.Sampling Locations Distribution

Table S1. Sampling Locations Distribution		
Sample Number	Gestational Week	Location
A1	11	Right Medulla Oblongata
A2	11	Left Medulla Oblongata
A3	11	Right Pons
A4	11	Left Pons
A5	11	Right Midbrain
A6	11	Left Midbrain
B2	12	Left Medulla Oblongata
B3	12	Left Pons
B4	12	Right Pons
B5	12	Left Midbrain
B6	12	Right Midbrain
C1	18	Medulla Oblongata
C2	18	Left Pons
C3	18	Right Pons
C4	18	Midbrain
D1	16	Medulla Oblongata
D2	16	Right Pons
D3	16	Left Pons
E1	28	Left Pons
E2	28	Left Midbrain
E3	28	Right Pons
F1	14	Right Pons
F2	14	Right Midbrain
F3	14	Left Pons

Table S2 continued.

Table S2. Sampling Locations Distribution		
Sample Number	Gestational Week	Location
F4	14	Left Midbrain
H1	17	Right Upper Midbrain
H2	17	Left Upper Midbrain
H3	17	Middle Of The right Midbrain
H4	17	Middle Of The Left Midbrain
H5	17	Lower Central Part Of The Midbrain
H6	17	Right Upper Pons
H7	17	Upper Central Part Of The Midbrain
H8	17	Left Upper Pons
H9	17	Right Lower Pons
H10	17	Left Lower Pons
H11	17	Lower Middle Part Of Pons
H13	17	Right Medulla Oblongata
H14	17	Left Medulla Oblongata
H15	17	Central Part Of The Medulla Oblongata
I1	11	Medulla Oblongata
I2	11	Pons
I3	11	Midbrain
J1	12	Medulla Oblongata
J3	12	Right Pons
J4	12	Left Midbrain
J5	12	Right Midbrain
K1	17	Left Upper Midbrain

Table S2 continued.

Table S2. Sampling Locations Distribution		
Sample Number	Gestational Week	Location
K2	17	Right Upper Midbrain
K3	17	Middle Part Of The Midbrain
K4	17	Lower right Part Of The Midbrain
K5	17	Lower Left Part Of The Midbrain
K6	17	Central Upper Part Of The Pons
K7	17	Right Upper Part Of The Pons
K8	17	Left Upper Part Of The Pons
K9	17	Right Middle Part Of The Pons
K10	17	Middle-to-lower Part Of The Pons
K11	17	Middle Part Of The Medulla Oblongata
K12	17	Right Medulla Oblongata
K13	17	Middle-to-lower Part Of The Medulla Oblongata
K14	17	Lower Part Of The Medulla Oblongata
L1	11	Medulla Oblongata
L3	11	Pons
L4	11	Midbrain
M1	13	Right Midbrain
M2	13	Left Midbrain
M3	13	Upper Part Of The Pons
M4	13	Lower Part Of The Pons
M5	13	Medulla Oblongata
M6	13	Lower Part Of The Medulla Oblongata
N1	12	Right Midbrain

Table S2 continued.

Table S2. Sampling Locations Distribution		
Sample Number	Gestational Week	Location
N2	12	Left Midbrain
N3	12	Upper Part Of The Pons
N4	12	Middle Part Of The Pons
N5	12	Medulla Oblongata
R1	11	Left Medulla Oblongata
R2	11	Right Medulla Oblongata
R3	11	Left Pons
R4	11	Right Pons
R5	11	Left Midbrain
R6	11	Right Midbrain
S1	9	Right Midbrain
S2	9	Left Midbrain
S3	9	Pons
S4	9	Medulla Oblongata
T1	12	Right Midbrain
T2	12	Left Midbrain
T3	12	Pons
T4	12	Medulla Oblongata
U1	33	Right Midbrain
U2	33	Left Midbrain
U3	33	Right Pons
U4	33	Left Upper Pons
U5	33	Left Lower Pons

Table S2 continued.

Table S2. Sampling Locations Distribution		
Sample Number	Gestational Week	Location
U6	33	Left Medulla Oblongata
U7	33	Right Medulla Oblongata
V1	24	Central Lower Part Of The Medulla Oblongata
V2	24	Middle Part Of The Left Medulla Oblongata
V3	24	Middle Part Of The right Medulla Oblongata
V4	24	Upper Part Of The Left Medulla Oblongata
V5	24	Upper Part Of The right Medulla Oblongata
V6	24	Left Upper Midbrain
V7	24	Right Upper Midbrain
V8	24	Middle Part Of The Midbrain
V9	24	Anterior-superior Part Of The Basal Region Of The Left Pons
V10	24	Lower Part Of The Basal Region Of The Left Pons
V11	24	Lower Part Of The Tegmental Region Of The Left Pons
V12	24	Right Pons

Table S2 summarizing fetal brainstem sampling details for RNA-seq, including sample IDs, gestational ages (GW), and anatomical locations. Letters denote individual fetuses (n=18 fetuses from 11 GWs), with 107 total locations sequenced.

Table S3.Gene Modules

Table S3A

**Module: black (Size: 192) Gene Ontology Terms**
limb bud formation
negative regulation of protein autoubiquitination
negative regulation of intracellular steroid hormone receptor signaling pathway
H3K27me3 modified histone binding
neurotransmitter transmembrane transporter activity
inward rectifier potassium channel activity
positive regulation of BMP signaling pathway
embryonic digestive tract development
histamine secretion
histamine secretion by mast cell

Table S3B

**Module: blue (Size: 852) Gene Ontology Terms**
signaling receptor activity
defense response to protozoan
testosterone dehydrogenase (NAD+) activity
transmembrane signaling receptor activity
response to protozoan
negative regulation of tyrosine phosphorylation of STAT protein
immunoglobulin production
serotonin receptor signaling pathway
G protein-coupled serotonin receptor signaling pathway
regulation of IRE1-mediated unfolded protein response

Table S3C

Module: brown (Size: 488) Gene Ontology Terms
regulation of cyclin-dependent protein serine/threonine kinase activity
neuron migration
cytokine receptor activity
MHC class II protein binding
phosphate-containing compound metabolic process
negative regulation of peptide secretion
cyclin-dependent protein serine/threonine kinase regulator activity
calcium channel complex
cellular response to lithium ion
T-tubule

Table S3D

Module: cyan (Size: 99) Gene Ontology Terms
extracellular matrix structural constituent
collagen-containing extracellular matrix
extracellular region
extracellular matrix
collagen trimer
endoplasmic reticulum lumen
cell adhesion
extracellular matrix organization
extracellular structure organization
cell periphery

Table S3E

Module: darkred (Size: 17) Gene Ontology Terms
septin ring assembly
trophectodermal cell fate commitment
positive regulation of activation of membrane attack complex
oviduct epithelium development
positive regulation of growth rate
oviduct development
ureteric peristalsis
cardiac jelly development
outer hair cell apoptotic process
gamma-butyrobetaine dioxygenase activity

Table S3F

Module: green (Size: 482) Gene Ontology Terms
steroid hydroxylase activity
small GTPase binding
histone pre-mRNA DCP binding
GTPase binding
skin epidermis development
Golgi to lysosome transport
hair follicle development
regulation of stem cell differentiation
hair cycle
hair cycle process

Table S3G

Module: greenyellow (Size: 45) Gene Ontology Terms
tetrahydrofolate interconversion
tetrahydrofolate metabolic process
one-carbon metabolic process
folic acid-containing compound metabolic process
positive regulation of cytosolic calcium ion concentration involved in phospholipase C-activating G protein-coupled signaling pathway
oxidoreductase activity, acting on the CH-NH group of donors
pteridine-containing compound metabolic process
olfactory bulb development
regulation of TOR signaling
olfactory lobe development

Table S3H

Module: grey (Size: 107) Gene Ontology Terms
regulation of heart contraction
animal organ development
circulatory system development
heart contraction
heart process
tissue development
thyroid hormone binding
system development
anatomical structure development
cell differentiation

Table S3I

Module: grey60 (Size: 19) Gene Ontology Terms
monocyte activation involved in immune response
negative regulation of chemokine (C-X-C motif) ligand 9 production
CDP-diacylglycerol-glycerol-3-phosphate 3-phosphatidyltransferase activity
extracellular matrix constituent, lubricant activity
NADHX epimerase activity
rRNA (uridine-C5-)-methyltransferase activity
extracellular matrix
protein deamination
negative regulation of chemokine (C-X-C motif) ligand 2 production
ErbB-2 class receptor binding

Table S3J

Module: lightcyan (Size: 37) Gene Ontology Terms
cellular response to low-density lipoprotein particle stimulus
myotube differentiation
regulation of macrophage migration
regulation of myotube differentiation
antibacterial peptide biosynthetic process
spermidine transport
cellular response to amiloride
sensory perception of salty taste
negative regulation of VCP-NPL4-UFD1 AAA ATPase complex assembly
growing cell tip

Table S3K

Module: lightgreen (Size: 15) Gene Ontology Terms
maturation of SSU-rRNA from tricistronic rRNA transcript (SSU-rRNA, 5.8S rRNA, LSU-rRNA)
small-subunit processome
maturation of SSU-rRNA
protein import into peroxisome matrix, substrate release
trachea cartilage development
head development
preribosome
growth plate cartilage chondrocyte growth
traversing start control point of mitotic cell cycle
protein import into peroxisome matrix, docking

Table S3L

Module: lightyellow (Size: 11) Gene Ontology Terms
gonad morphogenesis
phytosphingosine biosynthetic process
spinal cord interneuron axon guidance
dorsal spinal cord interneuron anterior axon guidance
regulation of cellular response to testosterone stimulus
zinc efflux transmembrane transporter activity
phytoceramidase activity
interneuron axon guidance
androgen binding
ATP-activated inward rectifier potassium channel activity

Table S3M

Module: magenta (Size: 83) Gene Ontology Terms
regulation of mismatch repair
transition metal ion binding
alkaloid metabolic process
importin-alpha family protein binding
heart valve morphogenesis
toxin metabolic process
photoreceptor outer segment membrane
response to morphine
heart valve development
heart formation

Table S3N

Module: midnightblue (Size: 42) Gene Ontology Terms
DNA catabolic process, endonucleolytic
humoral immune response
positive regulation of B cell differentiation
serine-type endopeptidase activity
DNA catabolic process
serine-type peptidase activity
homeostatic process
serine hydrolase activity
regulation of B cell differentiation
positive regulation of organ growth

Table S3O

Module: pink (Size: 128) Gene Ontology Terms
peptide catabolic process
negative regulation of neural precursor cell proliferation
putrescine metabolic process
positive regulation of execution phase of apoptosis
negative regulation of exocytosis
nicotinamide nucleotide metabolic process
basolateral plasma membrane
negative regulation of neuroblast proliferation
positive regulation of phagocytosis, engulfment
positive regulation of CD8-positive, alpha-beta T cell activation

Table S3P

Module: purple (Size: 86) Gene Ontology Terms
sperm head plasma membrane
oxidative DNA demethylase activity
sperm head
protein tyrosine phosphatase activity
phosphoprotein phosphatase activity
pi-body
bleb
phosphatidylinositol-3-phosphate phosphatase activity
phosphatidylinositol-3,5-bisphosphate 3-phosphatase activity
piRNA processing

Table S3Q

Module: red (Size: 468) Gene Ontology Terms
T cell activation
alpha-beta T cell proliferation
positive regulation of immune response
peptidyl-tyrosine autophosphorylation
early phagosome
adaptive immune response
alpha-beta T cell activation
histone lysine methylation
T cell proliferation
histone H3-K4 methylation

Table S3R

Module: royalblue (Size: 19) Gene Ontology Terms
protein glycosylation
macromolecule glycosylation
glycosylation
signaling receptor activity
glycoprotein biosynthetic process
endochondral bone growth
positive regulation of smooth muscle cell migration
bone growth
glycoprotein metabolic process
cardiac epithelial to mesenchymal transition

Table S3S

Module: salmon (Size: 54) Gene Ontology Terms
intercellular bridge
lipid binding
plasma kallikrein-kinin cascade
activation of plasma proteins involved in acute inflammatory response
proepicardium cell migration involved in pericardium morphogenesis
positive regulation of platelet-derived growth factor receptor signaling pathway
fumarate transport
medial motor column neuron differentiation
canonical Wnt signaling pathway involved in neural crest cell differentiation
negative regulation of mitotic centrosome separation

Table S3T

Module: tan (Size: 49) Gene Ontology Terms
monoamine:proton antiporter activity
monoamine transmembrane transporter activity
serotonin uptake
serotonin transport
transport
clathrin-sculpted monoamine transport vesicle membrane
serotonin:sodium:chloride symporter activity
neurotransmitter reuptake
aminergic neurotransmitter loading into synaptic vesicle
antiporter activity

Table S3U

Module: turquoise (Size: 4571) Gene Ontology Terms
structural constituent of ribosome
protein-containing complex
cytoplasmic translation
cytosolic ribosome
ribosome
ribonucleoprotein complex
large ribosomal subunit
catalytic complex
cytosolic large ribosomal subunit
translation

Table S3V

Module: yellow (Size: 657) Gene Ontology Terms
female gamete generation
ovulation cycle process
ovulation
endosome lumen
embryonic viscerocranium morphogenesis
alpha-glucosidase activity
ovulation cycle
cell body membrane
inorganic phosphate transmembrane transporter activity
regulation of tube size

Table S3 (A-V) present the functional annotations associated with each of the 22 gene modules identified through WGCNA clustering.

Table S4.GSVA Scores

Table S3A.GSVA Scores for Group A

GeneSet	A1	A2	A4	A3	A6	A5
black	-0.39960403	-0.420597880	-0.40546248	-0.40414925	-0.42104612	-0.43932243
blue	-0.57072180	-0.566111972	-0.57258155	-0.56246788	-0.55736342	-0.57271875
brown	0.17611024	0.214765373	0.17692098	0.14968661	0.07765611	0.15178221
cyan	0.29255775	0.317986711	0.12046242	0.04090709	-0.18130120	0.21902327
darkred	0.18117605	0.180025315	-0.02593555	-0.06987403	-0.11542884	-0.01187676
green	-0.58267525	-0.585087074	-0.57856006	-0.59297334	-0.57809896	-0.57675756
greenyellow	-0.39785013	-0.332079763	-0.43175841	-0.41837172	-0.43128279	-0.44103524
grey	-0.07248141	-0.115982469	-0.06355590	-0.27121063	-0.04323208	0.14970154
grey60	0.09021407	-0.003175723	0.11632557	-0.05581926	0.11020936	0.10679840
lightcyan	0.04694055	0.023119134	0.07714613	-0.06657341	-0.06625197	-0.06443005
lightgreen	-0.15142253	-0.297907360	-0.20773572	-0.13660945	-0.15800611	-0.13225958
lightyellow	0.37286191	0.247639045	0.33035585	0.05784774	0.35816771	0.44323643
magenta	-0.13173627	-0.064950972	-0.13703781	-0.17903383	-0.28513910	-0.28183882
midnightblue	-0.32174471	-0.222707737	-0.30926962	-0.13292914	-0.07633632	-0.25597101
pink	-0.32432693	-0.299206116	-0.34034040	-0.31541456	-0.28230900	-0.30952563

Table S4A continued.

GeneSet	A1	A2	A4	A3	A6	A5
purple	-0.33956465	-0.417635816	-0.38542030	-0.36397307	-0.38571791	-0.40311878
red	-0.55651399	-0.568093541	-0.56290195	-0.55835132	-0.56341819	-0.57009318
royalblue	-0.11356193	-0.057969078	-0.07935263	-0.19414329	-0.23323924	-0.15572806
salmon	-0.32019908	-0.307404611	-0.37772043	-0.36549329	-0.31253206	-0.37894254
tan	0.07290990	0.056204196	0.03397988	0.10508237	0.09718606	0.03239023
turquoise	0.25976523	0.234916073	0.23392850	0.21138292	0.27863714	0.26514073
yellow	-0.33194833	-0.340297800	-0.32422794	-0.30597810	-0.33384326	-0.34371448

Table S4B.GSVA Scores for Group B

GeneSet	B2	B3	B4	B5	B6
black	-0.38708794	-0.382454559	-0.39246086	-0.404541886	-0.42653104
blue	-0.56784640	-0.578134435	-0.57508785	-0.559098938	-0.57111815
brown	0.15196159	0.168105035	0.15976379	0.086906391	0.15412205
cyan	0.12964998	0.015445352	-0.15545436	-0.218860454	-0.08850657
darkred	0.14992944	0.101481656	0.02551740	0.106221077	0.05422879
green	-0.58991838	-0.589221844	-0.59122231	-0.578787359	-0.57564900
greenyellow	-0.27184105	-0.323497424	-0.40204089	-0.357799282	-0.41143853
grey	-0.22857602	-0.265164637	-0.17228552	-0.031656094	0.04700490
grey60	0.10312931	0.154669490	0.05880969	-0.179487179	0.04645966
lightcyan	-0.04228947	-0.006577771	-0.13132961	-0.118166445	-0.06279694
lightgreen	-0.05878204	-0.095814719	-0.03444627	-0.001977525	-0.03658849
lightyellow	0.37804256	0.206815511	0.36113137	0.333412976	0.38856632

Table S4B continued.

GeneSet	B2	B3	B4	B5	B6
magenta	-0.15197967	-0.178806090	-0.27747947	-0.234792687	-0.25523093
midnightblue	-0.28373617	-0.219342914	-0.22927232	-0.159555248	-0.18218375
pink	-0.31737966	-0.323817940	-0.33229956	-0.286778552	-0.29702215
purple	-0.41528625	-0.417808804	-0.42852781	-0.395106562	-0.39671483
red	-0.56005263	-0.546277649	-0.56003945	-0.552338282	-0.56268820
royalblue	-0.16631381	-0.160903317	-0.21806634	-0.200658669	-0.26217361
salmon	-0.24628322	-0.259444683	-0.30648489	-0.266599184	-0.30470739
tan	-0.02031823	0.013716313	-0.03306795	0.072664398	0.03285531
turquoise	0.25129901	0.273611911	0.26386149	0.261412739	0.26594043
yellow	-0.32818622	-0.321714815	-0.33090414	-0.338604685	-0.34649821

Table S4C.GSVA Scores for Group C

GeneSet	C1	C2	C3	C4
black	-0.44850105	-0.47258540	-0.43975869	-0.45326620
blue	-0.52137798	-0.49391095	-0.53882264	-0.54884290
brown	0.12652147	0.12900028	0.11736513	0.16913823
cyan	0.80124584	0.93882960	0.81885393	0.61274386
darkred	0.47242671	0.35509056	0.33673047	0.22507056
green	-0.58303691	-0.53521608	-0.59967534	-0.58928208
greenyellow	-0.16568816	-0.04640651	-0.19316663	-0.30423640
grey	0.24683389	0.08605928	0.20213232	-0.12262492
grey60	0.44205623	0.31456672	0.36047384	0.03799106
lightcyan	0.14158784	0.13418802	0.15809820	0.07554690
lightgreen	0.07503911	0.38948029	0.06410744	0.11041831
lightyellow	0.34880990	0.32091341	0.25762125	0.01533525

Table S4C continued.

GeneSet	C1	C2	C3	C4
magenta	-0.21312288	-0.14484211	-0.22894768	-0.18533654
midnightblue	-0.12283453	-0.18569835	-0.03723002	-0.10993112
pink	-0.23868918	-0.25361762	-0.20586837	-0.30034003
purple	-0.18022123	-0.28596817	-0.24848350	-0.34943859
red	-0.55514004	-0.54807521	-0.57493556	-0.56969368
royalblue	0.02227927	0.21882694	0.05043272	-0.20370672
salmon	-0.38280385	-0.34851431	-0.36047289	-0.23434521
tan	0.38745029	0.30593149	0.29937105	0.01242979
turquoise	0.18770453	0.19117359	0.19154228	0.23013437
yellow	-0.25210405	-0.18158648	-0.27114414	-0.32714638

Table S4D.GSVA Scores for Group D

GeneSet	D1	D2	D3
black	-0.40385529	-0.45521909	-0.47394939
blue	-0.53911309	-0.53140849	-0.54134682
brown	0.17696377	0.16012439	0.10832748
cyan	0.14012270	0.65901684	0.72671111
darkred	-0.02379986	0.23259643	0.32972719
green	-0.57950514	-0.59378244	-0.60640901
greenyellow	-0.37065959	-0.25205818	-0.21519148
grey	-0.30559439	0.02653549	0.13959122
grey60	0.06943131	0.11173841	0.19724771
lightcyan	0.06221389	0.18015530	0.34627813
lightgreen	0.21132743	0.09985825	0.21015244

Table S4D continued.

lightyellow	0.15663925	0.52894747	0.33980885
magenta	-0.22929399	-0.11599827	-0.04296603
midnightblue	-0.09815422	-0.17462281	-0.03391742
pink	-0.27578122	-0.27509243	-0.28435324
purple	-0.30648604	-0.29967725	-0.36095671
red	-0.55096965	-0.56863132	-0.56817967
royalblue	0.04378981	-0.19340002	0.03570285
salmon	-0.22001136	-0.21534761	-0.38131174
tan	0.12017955	0.13863801	0.34743290
turquoise	0.21932392	0.20276664	0.22465487
yellow	-0.29241581	-0.26318995	-0.25770706

Table S4E.GSVA Scores for Group E

GeneSet	E1	E2	E3
black	-0.43693933	-0.43613174	-0.415605619
blue	-0.55345922	-0.56691385	-0.550852110
brown	0.11663629	0.11399308	0.109070542
cyan	0.11331258	0.36821377	0.162127847
darkred	-0.05926623	0.05056444	-0.133349012
green	-0.58341658	-0.56821411	-0.586064239
greenyellow	-0.37644049	-0.36133977	-0.324409113
grey	0.34463194	0.37463529	0.323266772
grey60	0.07873512	-0.01275929	0.060772996
lightcyan	0.01039110	0.18480152	0.034936532
lightgreen	0.39111498	0.24597859	0.324571747
lightyellow	0.10895532	0.28335495	0.124397969

Table S4E continued.

GeneSet	E1	E2	E3
magenta	-0.18096183	-0.21062503	-0.159154930
midnightblue	-0.08081639	-0.15513055	-0.162691622
pink	-0.30312933	-0.28841918	-0.269461500
purple	-0.34964776	-0.43827663	-0.413586879
red	-0.55603830	-0.55496352	-0.548885390
royalblue	-0.02528817	-0.15219948	-0.008598313
salmon	-0.29094785	-0.25447997	-0.275308373
tan	0.32608030	0.21777125	0.243114772
turquoise	0.21821971	0.22680801	0.220932326
yellow	-0.28465825	-0.28242472	-0.286601612

Table S4F.GSVA Scores for Group F

GeneSet	F1	F2	F3	F4
black	-0.23159842	-0.43892222	-0.382644686	-0.4542102
blue	0.67091091	0.71702676	0.705159856	0.7130345
brown	0.56747064	0.52440212	0.579399373	0.5200673
cyan	-0.09119698	-0.06375408	-0.093543085	-0.1175316
darkred	0.08359836	-0.51598788	-0.208327381	-0.4898382
green	-0.35199104	-0.42421853	-0.410220384	-0.3859518
greenyellow	0.49703172	0.48302272	0.714190368	0.1219104
grey	0.08924927	0.16699820	-0.005092985	0.1831811
grey60	-0.66414364	-0.53346804	-0.684119328	-0.6378735
lightcyan	-0.47785464	-0.54693438	-0.580847339	-0.5265764
lightgreen	-0.16958919	-0.31312710	0.010193984	-0.1076236
lightyellow	-0.72915790	-0.76368978	-0.639482961	-0.6366627

Table S4F continued.

GeneSet	F1	F2	F3	F4
magenta	0.16602560	0.53474461	-0.430044397	-0.1707091
midnightblue	-0.53450259	-0.39258593	-0.435217342	-0.3480755
pink	0.05735947	0.08344939	-0.424745820	-0.4504910
purple	-0.08959845	0.48905146	-0.307359792	0.6031157
red	0.64853841	0.46549189	0.592694683	0.4148430
royalblue	-0.15278370	0.28262889	0.115536271	0.4826413
salmon	-0.60250483	-0.57388947	-0.369122324	-0.6265076
tan	-0.45527401	-0.45106927	-0.330073182	-0.4657148
turquoise	-0.17241811	-0.18510213	-0.186198234	-0.1416845
yellow	-0.63280770	-0.57396463	-0.622734529	-0.5708648

Table S4G.GSVA Scores for Group H (Part 1 of 2)

GeneSet	H1	H2	H3	H4	H5	H6	H7	H8
black	0.35240829	0.62775891	0.47814199	0.118694841	0.0122195	0.02180152	-0.347601168	0.383474978
blue	0.34145472	-0.03794104	0.41859862	0.571656613	0.5972985	-0.09306058	-0.006300702	0.375330073
brown	0.33492960	0.01603489	0.23319379	0.451877545	0.4741926	-0.56101470	-0.536027465	-0.008242634
cyan	-0.19924482	-0.27869576	-0.32184169	-0.190398457	-0.1651486	-0.20655018	-0.102137541	-0.334475792
darkred	0.11662024	-0.03913185	-0.48805806	-0.543231107	-0.4915318	0.34121960	-0.106061579	0.077348540
green	0.09971741	0.56093779	0.43275501	-0.246250300	-0.2577027	0.37160633	-0.031958271	0.431704666
greenyellow	0.40681735	0.65902017	0.89381784	0.951746107	0.9165435	-0.13729320	0.270965009	0.755306384

Table S4G continued.

GeneSet	H1	H2	H3	H4	H5	H6	H7	H8
grey	-0.06714062	-0.07731274	-0.11266241	-0.176875770	-0.1477120	0.08571662	0.158101710	-0.068124135
grey60	0.36216228	0.36154580	-0.42736898	-0.502134717	-0.4356657	0.98353329	0.567244011	0.368346820
lightcyan	-0.37906899	-0.37919081	-0.46769090	-0.528016210	-0.5948050	0.74818856	0.888784599	-0.200183965
lightgreen	-0.35573982	-0.13954855	-0.27406459	-0.369969545	-0.3598313	0.11429052	0.269909788	-0.162120856
lightyellow	-0.41180619	-0.46388295	-0.43695286	-0.539047134	-0.5624175	0.62984574	0.728008155	-0.036435182
magenta	0.71027909	0.58670178	0.05076739	-0.097994830	-0.1097151	0.70879336	-0.402975639	-0.227827913
midnightblue	-0.14228868	-0.28964415	-0.42689625	-0.421872903	-0.3912243	0.64203523	0.986672957	0.075462606
pink	0.55645954	0.56823170	0.12675612	0.004949648	0.1923317	0.37390928	-0.046058067	0.451603030
purple	-0.48053557	-0.60989467	-0.47788347	-0.522317615	-0.4897764	0.26165779	0.266598955	-0.399705976
red	0.66324224	0.66350625	0.57428616	0.594823910	0.6036111	-0.26623257	-0.394487314	0.505073357
royalblue	-0.40558632	-0.56837477	-0.50854206	-0.430209791	-0.3908264	0.09103740	0.298357571	-0.320576090
salmon	0.39361722	0.51494641	0.42912367	0.331469784	0.1271834	0.80329484	0.781469558	0.681436574
tan	-0.49668749	-0.42754524	-0.37427086	-0.256808493	-0.2805277	-0.16607456	0.239504110	-0.334208054
turquoise	-0.15585375	-0.12445351	-0.11715594	-0.147102233	-0.1650774	-0.12866533	-0.083517955	-0.237058841
yellow	-0.47605769	-0.39903899	-0.48354334	-0.561250794	-0.5711144	0.58328314	0.623361266	0.164714504

Table S4H.GSVA Scores for Group H (Part 2 of 2)

GeneSet	H9	H10	H11	H13	H14	H15
black	-0.311237509	-0.07071014	0.4034503	-0.277991434	0.5470321	-0.3221883209
blue	-0.208135617	-0.20554754	0.2540295	-0.186523827	-0.1022502	-0.2139650091
brown	-0.606841864	-0.58463138	0.2902998	-0.584214372	-0.1152905	-0.5830440611
cyan	0.119434262	-0.14225027	-0.2869531	-0.207021018	-0.3382032	0.0031845206
darkred	0.449384962	0.29659682	0.4801494	0.240801591	0.5183727	0.2753835211
green	0.165932053	0.26539129	0.1724403	0.301390351	0.4912403	0.0934930411
greenyellow	-0.349469765	0.26022112	0.5440624	-0.384177011	-0.1603214	-0.4166799404
grey	0.144460264	0.13692824	0.1032738	0.101355610	0.1353322	0.2148163556
grey60	0.682021237	0.68930709	0.7123542	0.603098680	0.2547655	0.3589163124
lightcyan	0.991041961	0.98703442	0.6759976	0.581164222	0.0471197	0.5453799507
lightgreen	0.012729786	-0.15647868	-0.3541860	0.170404625	-0.3429524	0.2842897020
lightyellow	0.704293636	0.77626322	0.2848738	0.604172293	-0.1409761	0.7756756757
magenta	-0.315368550	-0.24342076	0.3189870	-0.510737319	0.6812671	-0.5406207551
midnightblue	0.891763730	0.80970178	0.4724635	0.714817643	0.3196390	0.4742685426
pink	0.092302452	0.27244011	0.5599737	0.009079244	0.7366609	-0.1948580833

Table S4H continued.

GeneSet	H9	H10	H11	H13	H14	H15
purple	0.126074376	0.11764248	-0.5690258	0.272834237	-0.3296705	0.4649083887
red	-0.471246352	-0.17410913	0.6795304	-0.452954983	0.6349787	-0.4550434918
royalblue	-0.065113654	-0.04292578	-0.4340670	0.211850635	-0.4131165	0.1059113408
salmon	0.739479025	0.88533597	0.7030724	0.748744375	0.5783690	0.9676972728
tan	0.569508897	0.27725831	-0.4248011	0.522429318	-0.3780656	0.6207495053
turquoise	-0.009649505	-0.12041201	-0.2634742	-0.087290960	-0.1939771	-0.0007786747
yellow	0.698256208	0.70976542	0.2385364	0.750719942	0.1695179	0.7208425457

Table S4I.GSVA Scores for Group I

GeneSet	I1	I2	I3
black	-0.48312231	-0.54751718	-0.33834751
blue	0.61270883	0.64553589	0.62459416
brown	0.25982958	0.30268167	0.33767056
cyan	-0.12391199	-0.23759422	-0.29546063
darkred	0.82928152	0.80192412	0.22050451
green	-0.34865671	-0.40563468	0.03314329
greenyellow	-0.29617637	-0.23241314	-0.21087324
grey	0.09322441	0.14835390	0.03814643
grey60	-0.24754754	0.21357562	-0.36568832
lightcyan	0.17077664	0.17140170	-0.15060714

Table S4I continued.

GeneSet	I1	I2	I3
lightgreen	-0.58980431	-0.53859325	-0.26663203
lightyellow	-0.75017626	-0.78249119	-0.77884841
magenta	-0.62358636	-0.56120407	-0.18931193
midnightblue	0.43830580	0.38286749	-0.10489175
pink	-0.23002332	-0.17124529	-0.36075231
purple	0.57003694	0.52939912	0.05272937
red	0.27479268	0.28830151	0.50205966
royalblue	-0.11960417	-0.03725112	-0.10583529
salmon	-0.36880701	-0.02785438	-0.26486587
tan	0.80355269	0.76901124	0.63252838
turquoise	-0.21402386	-0.22592408	-0.21023717
yellow	0.44173248	0.44075718	-0.00118984

Table S4J.GSVA Scores for Group J

GeneSet	J1	J3	J4	J5
black	0.49470088	0.51767230	0.625911323	0.23066256
blue	0.26729481	-0.28359010	-0.181789676	0.53696602
brown	0.23326152	-0.36592938	-0.159340864	0.47510148
cyan	-0.33648003	-0.24718935	-0.271361720	-0.31847398
darkred	0.46525815	0.98424271	0.271574633	0.32144508
green	0.51051206	0.64321585	0.652285579	0.16328949
greenyellow	-0.15293961	-0.50034525	-0.297267667	0.31803769
grey	0.09240461	-0.06540138	0.016617992	0.10057208
grey60	-0.05605026	-0.03443731	0.050242026	0.01635618
lightcyan	-0.06420999	0.03385597	-0.115785640	-0.20747017

Table S4J continued.

GeneSet	J1	J3	J4	J5
lightgreen	-0.29979099	-0.25376919	-0.007912381	-0.27561183
lightyellow	-0.30114464	-0.28390312	-0.353040166	-0.74556952
magenta	0.43771590	-0.48033564	0.212244294	0.19986806
midnightblue	-0.50803067	-0.22534861	-0.503312935	-0.52577843
pink	0.62232598	0.11898048	0.225754358	0.53746906
purple	0.05843176	0.10071279	-0.414206665	-0.26448214
red	0.63074892	0.40727579	0.615146670	0.67556622
royalblue	-0.55078931	-0.27531507	-0.562779407	-0.43176166
salmon	-0.59941797	-0.24861600	-0.358822180	-0.57610351
tan	-0.51799378	0.68361665	-0.260629734	-0.50966896
turquoise	-0.19997424	-0.10422362	-0.103096082	-0.23094303
yellow	-0.28878918	0.46088034	-0.058617099	-0.36584401

Table S4K.GSVA Scores for Group K (Part 1 of 2)

GeneSet	K1	K2	K3	K4	K5	K6	K7	K8
black	0.57820554	0.215759412	0.14692301	0.48205597	-0.05214947	0.39493905	0.465211358	0.49486708
blue	-0.43660825	-0.346412946	-0.44622709	-0.48187977	-0.35256331	-0.43809436	-0.457303845	-0.50119043
brown	-0.64133981	-0.671844008	-0.67867991	-0.68920539	-0.67180275	-0.65307879	-0.673873426	-0.67141163
cyan	-0.27724324	-0.252775840	-0.28648675	-0.23828401	-0.24918725	-0.18029048	-0.288849046	-0.21662886
darkred	0.04598551	0.141053369	0.15109626	0.10771402	0.15505311	0.29019425	0.176681995	0.21636934
green	0.76917736	0.681058544	0.72578094	0.76902151	0.62113875	0.73097200	0.760269686	0.76406921

Table S4K continued.

GeneSet	K1	K2	K3	K4	K5	K6	K7	K8
greenyellow	0.25964753	-0.492296966	-0.55057808	-0.53490962	-0.63061766	-0.48133865	-0.002940907	-0.35490722
grey	-0.05854180	0.243811001	-0.10239968	0.06777723	0.01575112	0.12347861	-0.101879921	-0.00482091
grey60	-0.41722623	0.092038027	-0.01462956	-0.04278326	0.32159143	-0.01255514	-0.319457354	-0.04186751
lightcyan	0.10055175	0.337686303	0.31307716	0.20330106	0.29068888	0.36566778	0.244941157	0.35877465
lightgreen	-0.17193568	0.936985657	0.21855312	0.61870344	0.38511656	0.97919116	0.626253823	0.44458500
lightyellow	0.07967098	-0.002305113	0.84484835	0.38327191	0.49930331	0.24504010	0.200375957	0.41831122
magenta	-0.36076182	-0.221436398	-0.51272308	-0.32447227	-0.52581966	-0.36716642	-0.453038247	-0.39813481
midnightblue	0.41799240	0.331548406	0.31346834	0.09818185	0.40401916	0.35281315	0.319508918	0.38245162
pink	-0.43791678	-0.365166283	-0.42193634	-0.37585740	-0.34057399	-0.40698696	-0.431135638	-0.36348127
purple	0.33769807	0.589258634	0.44225341	0.47631969	0.45995655	0.41070477	0.334993330	0.24733720
red	-0.12238464	-0.503522723	-0.55849551	-0.48366057	-0.55357386	-0.49763390	-0.400995020	-0.42430290
royalblue	-0.34365461	-0.010929142	-0.15720775	-0.01858386	-0.13953838	-0.25842232	-0.236641661	-0.20995060
salmon	0.35709597	0.383412932	0.52431724	0.54493795	0.46238097	0.45282276	0.408460931	0.50078398
tan	0.47118337	0.593260855	0.52292523	0.61510291	0.70231752	0.22412984	0.558522734	0.55222224
turquoise	-0.10131177	-0.078859935	-0.04518738	-0.04071264	-0.04382463	-0.01763813	-0.079809376	-0.04403202
yellow	0.69269838	0.725104080	0.76854794	0.73109932	0.81643508	0.65593976	0.715074877	0.73516273

Table S4L.GSVA Scores for Group K (Part 2 of 2)

GeneSet	K9	K10	K11	K12	K13	K14
black	0.54303076	0.09475838	0.06970498	0.12730516	0.342758806	0.08426051
blue	0.17941266	-0.43214332	-0.41982905	-0.43602014	-0.486881653	-0.41972378
brown	-0.26577615	-0.67348093	-0.67147915	-0.66697662	-0.679536303	-0.66435737
cyan	-0.17325759	-0.26555283	-0.24957255	-0.24937510	-0.232448913	-0.24197589
darkred	-0.36970304	0.19332079	0.09929374	0.09587352	0.479820264	0.16740761
green	0.64073550	0.72327302	0.69682370	0.72994439	0.730129046	0.68536084
greenyellow	-0.16849952	-0.60861637	-0.52211985	-0.60639142	-0.499666440	-0.59546399
grey	0.08702225	-0.12541654	0.04111634	-0.11025244	-0.057550425	-0.01837626
grey60	-0.45840036	-0.03502193	0.15501772	-0.12447801	0.253413587	0.04832840
lightcyan	-0.48383350	0.30516237	0.30113806	0.34492801	0.384901641	0.36855710
lightgreen	-0.13781877	0.21625506	0.73851340	0.21929243	0.479212424	0.63375356
lightyellow	-0.63843634	0.83407813	0.53844536	0.88357501	0.386268937	0.70396710
magenta	0.70215691	-0.50153375	-0.45857886	-0.47659705	-0.425620068	-0.51703156
midnightblue	-0.22692035	0.21978655	0.17658144	0.20577344	0.264215527	0.30079789
pink	0.13701762	-0.42638739	-0.42210197	-0.44549452	-0.340003425	-0.37709935

Table S4L continued.

GeneSet	K9	K10	K11	K12	K13	K14
purple	0.43404386	0.52204741	0.50924098	0.50187536	0.365119316	0.57069014
red	0.42460348	-0.55680533	-0.57184186	-0.56447892	-0.513571091	-0.58702584
royalblue	-0.07361210	-0.16179533	-0.21865443	-0.15618936	-0.289219870	-0.20171724
salmon	-0.46841082	0.42985512	0.52353507	0.46908701	0.465195598	0.50760667
tan	-0.50223557	0.55329532	0.54722330	0.40538698	0.398268369	0.56608073
turquoise	-0.04287455	-0.03380364	-0.02825275	-0.05106275	-0.006861018	-0.04179527
yellow	-0.40979060	0.77167216	0.77704466	0.77086403	0.766671507	0.77235560

Table S4M.GSVA Scores for Group L

GeneSet	L1	L3	L4
black	-0.34595801	-0.25761312	-0.26721075
blue	0.56936386	0.61893224	0.63495279
brown	0.30817127	0.49749317	0.52420285
cyan	-0.26027300	-0.28845420	-0.24251420
darkred	0.11056928	0.05232284	0.17438521
green	-0.26343637	-0.23478887	-0.33584909
greenyellow	0.15945431	0.42770615	0.49473591
grey	0.05850287	-0.04069497	0.02783454
grey60	0.19068498	-0.37802128	-0.46353828
lightcyan	0.08475604	-0.26974395	-0.44836469
lightgreen	-0.36639168	-0.22423215	-0.21370226

Table S4M continued.

GeneSet	L1	L3	L4
lightyellow	-0.73666275	-0.60540541	-0.56660220
magenta	-0.39943433	-0.25632077	-0.39036235
midnightblue	0.38538068	-0.28372824	-0.21115933
pink	-0.06255125	-0.26060874	-0.40001798
purple	-0.03999512	-0.49499596	-0.48960451
red	0.54366369	0.63402580	0.60132622
royalblue	-0.12854569	-0.34827061	-0.35093165
salmon	0.22855768	-0.44210660	-0.48969048
tan	0.55560425	-0.36880557	-0.24649008
turquoise	-0.24389909	-0.19385895	-0.16273058
yellow	0.37166133	-0.44362714	-0.51263570

Table S4N.GSVA Scores for Group M

GeneSet	M1	M2	M3	M4	M5	M6
black	0.11790111	-0.40266785	-0.34392907	-0.36575419	-0.286573935	-0.4713970
blue	0.61906241	0.57530093	0.66912956	0.70691524	0.613850674	0.4624700
brown	0.49714112	-0.19316687	0.41995346	0.42665638	0.244223680	-0.3561651
cyan	-0.17897904	-0.08498090	-0.08880536	-0.12248757	-0.169812036	-0.2119084
darkred	-0.15628803	0.11361823	-0.43387576	-0.29429261	0.194990618	-0.3701982
green	-0.07438142	0.17013997	-0.12773345	-0.17899075	-0.120045650	0.0722891
greenyellow	0.41818119	-0.48968214	-0.25112821	0.02326059	0.001314098	-0.4656941

Table S4N continued.

GeneSet	M1	M2	M3	M4	M5	M6
grey	-0.12028484	0.11042811	0.22034254	0.28660511	0.268031562	0.2323021
grey60	-0.62408907	-0.40050409	-0.38594521	0.68503715	0.317894742	0.5501377
lightcyan	-0.49205322	-0.10984415	-0.37127860	-0.39567098	-0.125249134	0.8028318
lightgreen	-0.37289394	-0.33758360	-0.15611908	-0.20920868	-0.211860093	-0.1532165
lightyellow	-0.76803760	-0.69807412	-0.74477086	-0.78162998	-0.718157339	-0.2113097
magenta	0.56766833	-0.38733982	-0.08125873	0.39897890	0.050214406	-0.2989259
midnightblue	-0.40535283	0.52967840	-0.51417722	-0.40926380	0.206442518	0.6984391
pink	0.12113907	-0.40191257	-0.52776920	-0.34188053	-0.216947811	-0.3695947
purple	-0.23151622	0.86402663	0.59329619	0.66724298	0.572492217	0.7104837
red	0.67850844	-0.24370993	0.40725664	0.42010558	0.409859867	-0.3450462
royalblue	0.49910003	0.85442801	0.46959357	0.59265263	0.572754966	0.1771044
salmon	-0.62128411	-0.32471697	-0.60869571	-0.64536659	0.558169583	-0.2632708
tan	-0.47061239	-0.36500083	-0.46279014	-0.47871226	-0.320204297	0.7902890
turquoise	-0.20380846	-0.08146821	-0.11034687	-0.18532426	-0.246260237	-0.2181498
yellow	-0.56595691	0.04092120	-0.52289505	-0.49627493	0.307983219	0.6719640

Table S4O.GSVA Scores for Group N

GeneSet	N1	N2	N3	N4	N5
black	-0.46207141	-0.39002036	-0.35186538	-0.40636863	-0.42512809
blue	0.61042303	0.64493900	0.63468925	0.63193229	0.66785278
brown	-0.08201828	0.12708918	0.14793259	0.07137198	0.22315920
cyan	-0.12410567	-0.06966365	-0.02343696	-0.02100111	-0.08558477
darkred	-0.55385615	-0.61682741	-0.55038058	-0.59297145	-0.61203308
green	-0.22378721	-0.21661246	-0.08174174	-0.18085431	-0.25123330
greenyellow	-0.20811833	-0.30972421	-0.24945745	-0.30205471	-0.23122334
grey	0.18196189	-0.03562824	-0.05950020	-0.03719838	0.07118221
grey60	0.66814359	-0.59569889	-0.64455449	-0.49486960	-0.55643845
lightcyan	-0.10215924	-0.36123146	-0.45875121	-0.43180590	-0.37506473
lightgreen	-0.29341505	-0.52294847	-0.45750205	-0.52108845	-0.38249050
lightyellow	-0.26116693	-0.61260240	-0.61673160	-0.60296921	-0.64841363
magenta	-0.30320064	-0.37205507	-0.31483467	-0.37066379	-0.33706374
midnightblue	0.15350115	0.22523925	0.05754630	0.11764265	0.09664451
pink	-0.42269303	-0.46583612	-0.45255291	-0.43643795	-0.43578917
purple	0.82572270	0.79750733	0.80882281	0.82901533	0.84091055
red	-0.08066103	-0.02444726	0.06289157	-0.05095189	0.06797982
royalblue	0.89349989	0.83968864	0.83550669	0.86844665	0.86323397
salmon	0.44827861	-0.34650313	-0.50298392	-0.36450483	-0.37231705
tan	-0.34708168	-0.30248894	-0.39812355	-0.35262202	-0.37265022
turquoise	-0.17165239	-0.07047163	-0.06534006	-0.05010041	-0.09743206
yellow	0.24402392	-0.38870262	-0.48707853	-0.40503595	-0.38773155

Table S4P.GSVA Scores for Group R

GeneSet	R1	R2	R3	R4	R5	R6
black	0.01821484	-0.42850704	-0.3668238	-0.23916639	-0.44768752	-0.335744915
blue	0.64180569	0.67653561	0.7065402	0.65198652	0.70444002	0.689960468
brown	0.54225525	0.35225049	0.5774234	0.55086366	0.52844314	0.535630402
cyan	-0.24191210	-0.09979247	-0.1196382	-0.16964002	-0.08756127	-0.124998459
darkred	-0.09399304	-0.49957701	-0.4540579	-0.40831110	-0.32347014	-0.310208880
green	-0.15738409	-0.32849518	-0.4711190	-0.39974331	-0.44250885	-0.366209344
greenyellow	0.67599429	0.26176917	0.3523305	0.44988677	0.17486351	0.255352329
grey	-0.06073759	0.19065055	0.1099320	0.09465151	0.13870840	-0.024438556
grey60	-0.42556219	0.78224536	-0.3162549	-0.31300849	-0.45146964	-0.421418567
lightcyan	-0.43873387	-0.14275180	-0.4393917	-0.39535905	-0.38852707	-0.403027505
lightgreen	-0.30614053	0.58761534	-0.1248384	0.55295276	-0.27204903	-0.260465614
lightyellow	-0.71351351	-0.61756290	-0.6177438	-0.65992949	-0.64124559	-0.675052835
magenta	0.19418174	0.51767910	-0.1981065	0.23188050	-0.16435031	0.002922301
midnightblue	-0.48631362	-0.27178210	-0.4952229	-0.49956960	-0.50097173	-0.472258231
pink	0.07506609	-0.39166732	-0.4163503	-0.26143097	-0.44868548	-0.384690721
purple	-0.44613848	0.47872372	-0.2276791	-0.37840011	-0.18447132	-0.107943790
red	0.69365191	0.29592440	0.5995762	0.66950953	0.49524138	0.554771112

Table S4P continued.

GeneSet	R1	R2	R3	R4	R5	R6
royalblue	-0.16720825	0.23994996	0.3536571	0.05187420	-0.04228249	0.011902799
salmon	-0.60076126	-0.52474836	-0.5122338	-0.56443594	-0.47682964	-0.524403087
tan	-0.45168699	-0.37557979	-0.3273473	-0.23672893	-0.29645941	-0.379961735
turquoise	-0.21907821	-0.17282189	-0.1590504	-0.17164040	-0.12207309	-0.157788258
yellow	-0.57988253	-0.28516785	-0.5838356	-0.56644722	-0.58507989	-0.607209597

Table S4Q.GSVA Scores for Group S

GeneSet	S1	S2	S3	S4
black	-0.47077605	-0.47332068	-0.393085955	-0.44996811
blue	0.70852887	0.72238449	0.020553404	0.71495092
brown	0.51402903	0.52890338	-0.529913764	0.58773535
cyan	-0.07697769	-0.10378055	-0.110844307	-0.07983221
darkred	0.26828450	-0.20188738	0.203500043	0.03773107
green	-0.38204087	-0.39209350	0.155009678	-0.37684828
greenyellow	0.02547559	0.15495703	-0.486014215	0.12499907
grey	0.06785818	0.07840238	0.107400786	0.02363005
grey60	0.31634163	-0.54699117	0.414525682	-0.60545278
lightcyan	-0.31934087	-0.51703271	0.434362183	-0.52544421
lightgreen	-0.38309854	-0.36612370	0.734969332	-0.33962255
lightyellow	-0.74253819	-0.70752056	0.202950466	-0.66540721
magenta	0.30871706	0.16797452	-0.553810120	-0.17079204

Table S4Q continued.

GeneSet	S1	S2	S3	S4
midnightblue	-0.39129919	-0.50974857	0.606466299	-0.52684704
pink	-0.38189470	-0.31656491	-0.270473704	-0.35958807
purple	0.41363992	0.13751695	0.695497316	-0.30467021
red	0.42238552	0.46248018	-0.551481609	0.54668364
royalblue	0.33738313	0.36143360	0.003966597	0.04581676
salmon	-0.61293146	-0.60188235	0.805215104	-0.55993575
tan	-0.40908496	-0.41990472	0.799539698	-0.37813683
turquoise	-0.17315816	-0.16697469	-0.068976824	-0.15921050
yellow	-0.51370189	-0.57976224	0.703308348	-0.60222283

Table S4R.GSVA Scores for Group T

GeneSet	T1	T2	T3	T4
black	-0.265361690	-0.24100165	-0.52469421	-0.20611480
blue	0.642533881	0.67818754	0.23006630	0.66463897
brown	0.583418326	0.57608229	-0.50843414	0.55445570
cyan	-0.193975409	-0.13099126	-0.05925949	-0.12187400
darkred	0.090617887	0.04903574	0.29125170	-0.07307033
green	-0.359720975	-0.39710795	-0.12728006	-0.29023156
greenyellow	0.449364280	0.46225920	-0.48499067	0.23809791
grey	0.006631156	0.08901558	0.24344282	0.07940802
grey60	-0.288605376	-0.45513609	0.49983273	-0.38014595
lightcyan	-0.282479188	-0.38423532	0.57460479	-0.31015700
lightgreen	0.024914952	-0.14975783	0.67771623	-0.24891154
lightyellow	-0.608500526	-0.71930898	0.10472155	-0.68858970
magenta	-0.293539343	0.18643968	-0.22730762	0.18302432

Table S4R continued.

GeneSet	T1	T2	T3	T4
midnightblue	-0.299450927	-0.44151074	0.75323090	-0.48841683
pink	-0.124297951	0.13349272	-0.24915864	0.18170663
purple	-0.438228609	-0.43190668	0.70743931	-0.33740741
red	0.637595366	0.64769962	-0.53368726	0.66344047
royalblue	-0.421483274	-0.36936425	0.14679432	-0.24571670
salmon	-0.446957105	-0.51619244	0.40417281	-0.40665238
tan	-0.449010206	-0.43686505	0.87744493	-0.45136620
turquoise	-0.194669973	-0.20144717	-0.09741507	-0.20874801
yellow	-0.494793212	-0.56228945	0.67223316	-0.57052662

Table S4S.GSVA Scores for Group U

GeneSet	U1	U2	U3	U4	U5	U6	U7
black	0.742372465	0.83512635	0.82173695	0.79425241	0.864951410	0.83636285	0.850037958
blue	-0.261164588	-0.46190971	-0.34258578	-0.34250068	-0.508645959	-0.39638723	-0.400055317
brown	-0.528869529	-0.54388965	-0.33909597	-0.29815761	-0.536884046	-0.42874308	-0.476438298
cyan	-0.248869966	-0.17847932	-0.28727412	-0.22694216	-0.115187426	-0.32059842	-0.289347694
darkred	-0.307894017	-0.07402884	-0.38297792	-0.33744722	0.151567680	-0.38354320	-0.337356007
green	0.677359028	0.77466270	0.67890224	0.68535935	0.745811460	0.73865912	0.742423937
greenyellow	-0.561578740	-0.38977321	-0.08724340	-0.09128981	-0.351863445	-0.38264475	-0.386431365
grey	-0.003240877	0.02214300	-0.07675239	-0.08151892	0.085936076	-0.10503530	-0.104635397

Table S4S continued.

GeneSet	U1	U2	U3	U4	U5	U6	U7
grey60	-0.093705711	-0.10356035	0.16372580	-0.16351082	0.247415070	-0.15319529	-0.088202145
lightcyan	-0.348494473	-0.30653565	-0.27416122	-0.32757689	0.011467440	-0.38655144	-0.362575867
lightgreen	-0.074535735	0.15084662	0.19891361	0.16075358	0.352849962	0.14347472	0.157384382
lightyellow	-0.579338809	0.10316282	-0.18857418	-0.29675156	0.427225012	0.08897573	0.137845479
magenta	0.924463276	0.67314608	0.55938971	0.42585364	0.520165092	0.71679046	0.728692571
midnightblue	-0.106743605	-0.21663614	-0.40453782	-0.38412531	-0.120612352	-0.32017253	-0.300924401
pink	0.478355714	0.57880198	0.12568151	-0.07367111	0.548510778	0.37696597	0.458563285
purple	0.178325579	-0.47751570	-0.61996766	-0.60878662	-0.498429581	-0.57789275	-0.577041584
red	0.103168068	0.40606739	0.60286181	0.60627071	0.404412213	0.53773545	0.485900371
royalblue	0.151083247	-0.06227093	0.04262372	0.04292123	-0.099393416	0.11957937	0.004671504
salmon	-0.097685215	0.21917617	0.51255188	0.29810581	0.585299274	0.16196885	0.153548709
tan	-0.296731345	0.06563929	-0.18066186	-0.14460423	0.256237703	-0.23552511	-0.283808660
turquoise	0.043382576	0.02549917	-0.03668619	-0.02246507	-0.008821412	-0.02442034	-0.002384976
yellow	-0.182707295	-0.00438960	-0.18314707	-0.26863346	0.156069242	-0.23961702	-0.186372132

Table S4T.GSVA Scores for Group V (Part 1 of 2)

GeneSet	V1	V2	V3	V4	V5	V6	V7	V8
black	0.37722514	0.32612691	0.38416147	0.36078941	0.37639882	0.476986069	0.52633729	0.48384816
blue	0.26169531	0.39057741	0.33126611	0.36440939	0.36773998	0.457034588	0.41131925	0.44171128
brown	0.18878399	0.39691602	0.39008599	0.41464381	0.39793607	0.461992497	0.41777118	0.49222634
cyan	-0.23051594	-0.26533514	-0.26592631	-0.22946017	-0.21084619	-0.207108588	-0.26445435	-0.18511650
darkred	0.29980715	0.43662359	0.47851982	0.42432913	0.18737510	-0.436172644	-0.28155444	-0.41761853
green	0.35806883	0.07747016	0.20963076	0.14079523	0.02610028	0.006017307	0.16318514	0.03698790
greenyellow	0.16629646	0.23350655	0.09156906	0.29679001	0.37515486	0.949786828	0.94536945	0.94926852
grey	-0.03656023	-0.02709613	-0.13253498	-0.10447961	0.09094989	-0.119240758	-0.18050309	-0.15535026
grey60	-0.07381504	-0.15935599	-0.24869260	-0.35060527	-0.04813293	-0.478899170	-0.50635284	-0.55519426
lightcyan	-0.07705274	-0.16395792	-0.17879455	-0.20148838	-0.14048116	-0.334106842	-0.36260739	-0.39656104
lightgreen	-0.21027204	-0.20010225	-0.21805262	-0.28405189	-0.23326245	0.193766799	0.10110510	-0.09571084
lightyellow	0.32359939	0.01601503	0.15349055	-0.02503692	-0.38507377	0.067311591	0.06320230	-0.46351019
magenta	0.16030564	-0.03589588	-0.04154225	-0.13273476	0.58999043	0.027303109	0.04837736	-0.09898613
midnightblue	-0.27162306	-0.30305383	-0.30776649	-0.33546968	-0.28763269	-0.439856979	-0.46547617	-0.52898784
pink	0.89051417	0.90505008	0.86487059	0.87088643	0.85968297	0.643851416	0.66137804	0.51276663

Table S4T continued.

GeneSet	V1	V2	V3	V4	V5	V6	V7	V8
purple	-0.10519792	-0.40209192	-0.39427837	-0.37166909	-0.46143707	-0.568931383	-0.57727288	-0.60096403
red	0.44687799	0.58706930	0.58165340	0.61573408	0.63184510	0.669029207	0.65847499	0.67737726
royalblue	-0.58695490	-0.55813416	-0.49942667	-0.52286624	-0.41773094	-0.378545884	-0.59205379	-0.55126630
salmon	-0.24953489	-0.45572942	-0.47712747	-0.47779921	-0.52397469	-0.238439235	-0.29368425	-0.37405409
tan	-0.21669280	-0.29896308	-0.33031743	-0.31291619	-0.33991087	-0.345197103	-0.42790521	-0.28500103
turquoise	-0.16814725	-0.18986168	-0.17503315	-0.16748597	-0.22406339	-0.202537352	-0.17560400	-0.16431113
yellow	-0.26897690	-0.38604024	-0.36867455	-0.40330238	-0.41803734	-0.500689542	-0.49794365	-0.53304694

Table S4U.GSVA Scores for Group V (Part 2 of 2)

GeneSet	V9	V10	V11	V12
black	0.32657789	0.445762490	0.38697493	0.49199338
blue	0.49720164	0.438087187	0.51995991	0.22673057
brown	0.53647215	0.480447808	0.50983404	0.33523849
cyan	-0.15089859	-0.234057339	-0.24743779	-0.21791699
darkred	-0.38397614	-0.259202344	-0.33724901	0.11720983
green	-0.22682285	0.067705082	-0.01779709	0.30585018
greenyellow	0.96059462	0.930509674	0.89009761	0.20959020
grey	-0.12986103	-0.057912602	0.13322309	0.05387441
grey60	-0.55613278	-0.564219527	-0.51964617	-0.01020658
lightcyan	-0.39954845	-0.401174743	-0.42742128	-0.18290514
lightgreen	-0.13787606	0.003185183	-0.24542237	-0.25343174

Table S4U continued.

GeneSet	V9	V10	V11	V12
lightyellow	-0.63834809	-0.608068842	-0.68029476	-0.45382322
magenta	-0.06057656	-0.043156971	-0.10898424	0.36257704
midnightblue	-0.52217588	-0.451460507	-0.43474109	-0.29995387
pink	0.55648569	0.521473971	0.71705077	0.78631777
purple	-0.56110794	-0.573507818	-0.40519364	-0.51110754
red	0.68110158	0.665993249	0.69711315	0.65132736
royalblue	-0.53902088	-0.561316055	-0.29039454	-0.44144974
salmon	-0.46386426	-0.453307948	-0.52594971	-0.44313108
tan	-0.25475963	-0.337861057	-0.38810163	-0.31535832
turquoise	-0.19009413	-0.167970028	-0.23583061	-0.19548384
yellow	-0.54557295	-0.521774422	-0.52925998	-0.34027938

Table S4 (A-U) present the GSVA scores of the 22 WGCNA-clustered gene modules across tissue samples from each sampling site.

Table S5.Mfuzz Clusters

Brain Region	Cluster	Gene Symbol
Midbrain	1	LOC112267876, MRAP2, POU6F2, WFIKKN2, SYT6, TAFA4, PRELID2, C1QL2, DIO3, EYA4, FGF10, SORCS3, EBF3, TCERG1L, GAD1, GATA3, SLC13A4, GBX2, ANGPT2, TRIB2, GRIN2D, SERPIND1, HLA-DRB4, BARHL2, LRRTM1, ASIC2, LMO1, NOMO3, MC4R, ZFHX3, NTS, OPRK1, PBX3, PCDH8, PNOC, BNC2, CASZ1, PRLR, PROX1, LHX9, NPFFR1, EBF2, SHOX2, TAC1, EIF3CL, KCNAB1, GALNT14, CLMP, SCRT2, DIRAS3, SLC22A8
	2	IGF2BP1, IGF2BP2, GADD45G, PHF21B, COL5A2, CRHBP, DCC, ARX, PI16, SULF1, OTP, SSBP2, FLRT3, CFAP126, AMH, DKK2, RCOR2, HMGB2, KCNA1, LRRC10B, MFAP2, MFAP4, MYB, PI15, PLAG1, FEV, PTGFR, ISLR2, SINHCAF, NEUROG2, NECAB1, SLC1A5, TH, TBC1D3, ZBBX, SCUBE1, ADGRV1, ITGA8, ADAMTSL1, LHX2
	3	PLCD3, SYNPO, C1QTNF1, FAM43A, CRYAB, CPNE9, ADRA2C, CX3CR1, APLNR, FAM153B, DDN, FLG, DAAM2, FBXO2, CD163L1, LAMC2, TRIM72, RASL12, CDK18, CYB5R2, PRRX1, PAK6, RASGRF1, SLC18A3, C3, C4A, ADAM33, DDO, CEACAM21, CPNE6, TJP2

Table S5 continued.

Brain Region	Cluster	Gene Symbol
Pons	1	PRG4, KLHL41, IGF2BP1, IGF2BP2, LDB3, SCN11A, DSC3, EFNB1, EFNB2, EMX2, FBN2, FGF10, FOXG1, VGLL2, TEX49, GAS2, GFRA3, C4orf54, FOXA2, MYBPC1, MYH2, MYL3, MYL4, NEB, NR4A2, PAX7, PITX3, PLAG1, BNC2, CASZ1, MCTP2, ACTA1, SINHCAF, SRL, DMRTA2, CXCR5, EBF2, SLC3A1, KLF5, TBX1, TEAD4, TFAP2C, NR2E1, TNNI1, TTN, EIF3CL, TBC1D3, VIL1, SCUBE1, KDM5D, FZD8, ADGRV1, MYPN, RUNX1, ANGPTL1, LDB2, LHX2, FOXP2, PCLAF
Pons	2	ARAP2, ZNF488, SYT2, TMEM125, NEU4, FAM43A, FAM163A, CPNE9, ADRB1, DGKG, CABP7, DRD5, FAM153B, DDN, FLG, HMCN2, PYDC1, FBXO2, GJB1, KCNH5, GPD1, GPR17, FAM153A, GRIN2C, PACSIN1, HPCA, HTR7, SYT10, STAC2, AQP6, KCNJ16, IYD, ANKRD34C, IRAG2, HAPLN4, MAG, MAS1, MBP, MOBP, MOG, DBX2, NEFH, NOS1, MLXIPL, PLLP, CDK18, CYB5R2, PLP1, MOCOS, HR, GABRQ, KCNQ5, KLK6, PAK6, GJC2, ERMN, CARNS1, ARHGAP22, RASGRF1, SCN4B, TMEM88B, NDST4, ETNPPL, ACTR3C, SLC12A1, SLC18A3, SLCO1A2, TNR, C4A, TTPA, TUBA4A, SEC14L6, UGT8, MYRF, FA2H, CALN1, SLC25A18, KISS1R, DDO, BCAS1, HSD17B6, CEACAM21, CD8B, GPR37L1
Pons	3	MPZL2, SIX2, LOC112267876, COL2A1, COL5A2, COL6A2, COL12A1, CDC42EP5, SIK1, PRSS35, DLX5, EYA4, PI16, FOXD1, PDZRN3, FOXD2, SULF1, MXRA5, MYOF, DKK2, ANGPT2, CDH19, GSTT1, HBE1, HLA-DRB4, HMGB2, LOX, LDLRAD2, NOMO3, MFAP2, MFAP4, MGP, NOX4, PI15, STK26, VIT, PLEKHA4, S100A4, SULT1E1, TGFBR3, TWIST1, COL14A1, IRX6, SOCS3, ADAMTSL1
Medulla	1	MPZL2, KLHL41, IGF2BP1, LZTS1, WFIKKN2, DSC3, EBF1, EFNB1, EMX2, OTP, EBF3, SLC13A4, C4orf54, SERPIND1, FOXA1, NOMO3, MID1, NR4A2, DACT1, PITX2, BNC2, SERTAD4, PTGFR, ISLR2, TNNI1, TTR, SLC22A8
Medulla	2	FST, LOC112267876, COL2A1, COL5A2, COL6A2, COL12A1, DIO3, SULF1, HBE1, HBG1, HLA-DRB4, LOX, MFAP2, MFAP4, MYB, MYL4, ACTA1, S100A4, EBF2, SHOX2, BMP6, SLN, CLMP, FZD8

Table S5 continued.

Brain Region	Cluster	Gene Symbol
Medulla	3	LOC102724788, ADAM28, PADI2, CMTM5, ARAP2, GPR62, C10orf90, ZNF488, CNGA3, CCDC163, CNP, TMEM125, NEU4, SLC31A2, NACC2, AIFM3, CHADL, CX3CR1, MMD2, FGF1, SIRT2, FBXO2, GATM, GJB1, GPD1, TMEM235, GPR17, HMSD, PRR18, NIPAL4, AQP6, KCNJ10, KCNJ16, KCNN2, CCDC9B, SNORC, ANKRD34C, MAG, MAL, MBP, MOBP, MOG, MX1, NOS1, CLDN11, P2RX7, PLLP, CDK18, CYB5R2, PHKG1, PLP1, PLXNB3, PMP2, P2RY13, GPR88, TREM2, HERC6, TMEM144, IL17RB, FERMT1, INAVA, HR, PCDHAC1, PRL, KLK6, LPAR5, GJC2, ERMN, CARNS1, COL20A1, PTPRH, PLEKHB1, RASGRF1, RGR, BDNF, S100B, SCN2B, TMEM88B, PIRT, RASSF10, P2RY12, BMP3, SLC6A11, SLCO1A2, SOX10, TF, TNR, C4A, TTPA, UGT8, MYRF, FA2H, CALB2, OGFRL1, MYH14, PAQR6, TLR10, CHST9, QRFPR, BBOX1, BCAS1, DNAH17, SLC4A4, ACY3, GPR37L1, CPNE6, GAL3ST1, CD38, ENTPD2

Table S5 summarizes the gene modules of midbrain, pons and medulla, which were clustered by Mfuzz based on temporal expression patterns, and lists the genes contained in each module.

Table S6.Immunofluorescence Antibody

Gene	Antibody	Product ID	Company	RRID
C4A	Complement C4A Rabbit mAb	A3545	ABclonal	AB_3096350
MAP2	MAP2 Mouse Monoclonal Antibody	67015-1-Ig	proteintech	AB_2882331
DRD5	DRD5 Polyclonal antibody	20310-1-AP	proteintech	AB_10699880
NeuN	NeuN (D4G4O) XP® Rabbit mAb	24307S	CST	AB_2799470
NEFH	NF-H/NF200 Polyclonal antibody	18934-1-AP	proteintech	AB_10640801
TUBA4A	Anti-alpha Tubulin antibody	bsm-33039M	Bioss	AB_2864778

Table S6 presents the immunofluorescence antibody types employed for protein expression verification.
